# Flexible Unicast-Based Group Communication for CoAP-Enabled Devices ^†^

**DOI:** 10.3390/s140609833

**Published:** 2014-06-04

**Authors:** Isam Ishaq, Jeroen Hoebeke, Floris Van den Abeele, Jen Rossey, Ingrid Moerman, Piet Demeester

**Affiliations:** 1 Department of Information Technology (INTEC), Ghent University - iMinds, Gaston Crommenlaan 8 Bus 201, Ghent 9050, Belgium; E-Mails: jeroen.hoebeke@intec.ugent.be (J.H.); floris.vandenabeele@intec.ugent.be (F.V.A.); jen.rossey@intec.ugent.be (J.R.); ingrid.moerman@intec.ugent.be (I.M.); piet.demeester@intec.ugent.be (P.D.); 2 Said Khoury IT Center of Excellence (SKITCE), Al-Quds University, Abu Deis, Jerusalem 51000, Palestine

**Keywords:** Internet of Things, CoAP, sensors, wireless sensor networks, group communication, entities

## Abstract

Smart embedded objects will become an important part of what is called the Internet of Things. Applications often require concurrent interactions with several of these objects and their resources. Existing solutions have several limitations in terms of reliability, flexibility and manageability of such groups of objects. To overcome these limitations we propose an intermediately level of intelligence to easily manipulate a group of resources across multiple smart objects, building upon the Constrained Application Protocol (CoAP). We describe the design of our solution to create and manipulate a group of CoAP resources using a single client request. Furthermore we introduce the concept of profiles for the created groups. The use of profiles allows the client to specify in more detail how the group should behave. We have implemented our solution and demonstrate that it covers the complete group life-cycle, *i.e.*, creation, validation, flexible usage and deletion. Finally, we quantitatively analyze the performance of our solution and compare it against multicast-based CoAP group communication. The results show that our solution improves reliability and flexibility with a trade-off in increased communication overhead.

## 1. Introduction

The Do-It-Yourself (DIY) movement is spreading beyond traditional domains, such as home painting, to more modern domains, such as programming. DIY programming gets especially interesting when it involves real-time data from the growing amount of smart objects with embedded sensors and when actuators can be triggered to perform real-world actions accordingly. It becomes even more interesting and appealing when access to these smart objects can be obtained over the ubiquitous Internet—leading to what is now mostly known as the Internet of Things (IoT). However, these smart objects are typically optimized for low-power consumption and low-cost. They are constrained in their processing capabilities (CPU, RAM, ROM,…) and thus unable to run standard Internet protocols. The networks that connect these objects together are often referred to as low power and lossy networks (LLNs).

Connecting LLNs to the Internet, communicating with smart objects, and manipulation of sensor data and actuators was largely done in proprietary ways. Each vendor had its own set of protocols and tools to access, interpret and if needed manipulate sensor data and to trigger actuators. More recently a lot of effort has been put into the development of open standards that cover many aspects of communication and access to smart objects. At the networking layer 6LoWPAN allows IPv6 communication with these objects through an adaptation layer [2]. At the application layer standards are being prepared to allow access to these objects in a RESTful way, similar to how most information on today's Internet is accessed over HTTP. The main driver behind this is the Internet Engineering Task Force (IETF). The IETF established the Constrained RESTful Environments (CoRE) working group with the aim of realizing the REST architecture in a suitable form for the most constrained nodes and networks. Constrained devices are turned into embedded web servers that make their resources accessible via the CoAP protocol. CoRE is aimed at machine-to-machine (M2M) applications such as smart energy and building automation [3].

Typically, each of the constrained servers has at least one CoAP resource that may be queried by clients to obtain information about the smart objects themselves (e.g., battery level), about the environment that they monitor (e.g., temperature of the room), or to trigger the objects to perform real-world actions (switch the light on). These CoAP resources are identified by a Uniform Resource Identifier (URI) such as coap://[aaaa::1]/temperature.

Depending on the application, information from individual objects might not be sufficient, reliable, or useful. An application may need to aggregate and/or compare data from several nodes in order to obtain accurate results. In the same way, a single user request might need to trigger a series of actions on multiple actuators. This need to communicate with groups of objects is obvious in many IoT scenarios. For example, in a smart home, when you leave your bed during the night, you might want that the lights in the bedroom, hall and toilet turn on automatically until you go to bed again. Or, when suspicious movement is detected in the living area during the night, several actuators may be triggered such as an alarm going off and particular lights being turned on or made flashing. From these two simple examples, one can already see that the same lights can be parts of different groups according to the needs of the user. The needs can change regularly and thus the grouping and ungrouping of resources should be flexible and easy. Similar examples for the need of group communication can be found in virtually any IoT scenario.

The need for group communication is very well recognized in the IETF. This can be clearly seen from the charter of the IETF CoRE Working Group. The charter clearly states that the “initial work item of the WG is to define a protocol specification for CoAP that includes … the ability to support a non-reliable multicast message to be sent to a group of Devices to manipulate a resource on all the Devices in the group.” The charter also states that “the working group will not develop a reliable multicast solution” [4].

Although multicast may be used to transmit the same request to several objects, multicast communication in LLNs has some disadvantages. For instance, it is more difficult to route multicast traffic with a minimum of message duplication at the receiving hosts than in the case of unicast. Furthermore, basic multicast is not reliable in an LLN, which is problematic for requests that require guaranteed delivery. Also, the creation of multicast groups, defining which objects should be addressed when using a particular multicast address, is hard to realize inside LLNs. Additionally, the use of network wide multicast increases the footprint of the code that needs to fit on the constrained objects, and it is to be expected that this functionality will not be available in many LLNs.

As an alternative, unicast-based solutions may be considered. Some unicast-based solutions (such as reliable messaging) have been introduced to alleviate some of the problems above, but these features are insufficient. The current CoRE drafts do not foresee any unicast-based way to manipulate resources that are located on multiple smart objects with a single client request. To overcome this shortcoming and be able to perform such composite requests, intelligence is typically added to the client application to make it communicate with the smart objects individually. This leads to more complex user applications, and the added intelligence and programming cannot be easily shared with other applications. Furthermore, complex user applications may be unmanageable. Any modifications to those complex user applications may require significant testing time, thus limiting the flexibility of the user applications. Additionally a large overhead of communication between the client machine and the smart objects is generated, especially when many smart objects are involved in these actions. When the communication between the client and the smart objects is done across the Internet, delays are unpredictable and a sequence of actuator commands might arrive out of order and possibly have unwanted results. Furthermore, if the communication occurs over costly links, communication between the client and the smart objects might get unnecessarily expensive.

In this paper we propose a novel solution for communication with a group of resources across multiple smart objects based on CoAP unicast. The group members can be homogeneous or heterogeneous, on a single node or on multiple nodes, or another group. The group that we create is itself exposed as a RESTful CoAP resource, and thus can be accessed by any CoAP client (including other constrained devices). We include optional validation of the group at creation time; we attach a profile to the created group and thus can customize its behavior and provide fine-grained control over it. We have implemented our solution and provide a functional and performance evaluation for it. In the past, we have already presented our concept along with an initial implementation in [1]. In this expanded article, we elaborate on the concept, add more advanced features to the implementation, compare our solution with multicast and evaluate its functionality and performance.

The remainder of this paper is structured as follows: first, we will briefly provide an overview of CoAP in Section 2. We then discuss CoAP group communication requirements and related work in Sections 3 and 4. Next, in Section 5, we describe our approach in detail. In Section 6, we present our implementation and evaluate the functionally and the performance of our solution. In Section 7, we discuss the results and compare them to the requirements. Section 8 concludes this work with a summary and outlook.

## 2. CoAP Overview

The focus of this paper is to enable interaction with a group of devices from a service/application perspective in a way that is in line with ongoing standardization activities in the field of IoT. In the last few years a lot of effort has been put in defining a standard application protocol, similar to HTTP, but more suitable for constrained devices, namely CoAP. The base CoAP protocol is defined in draft-ietf-core-coap [5] in conjunction with a number of additional specifications. In this section we briefly introduce the base CoAP specification and those extensions that are relevant to our group communication work.

### 2.1. Base CoAP

**Figure 1.**
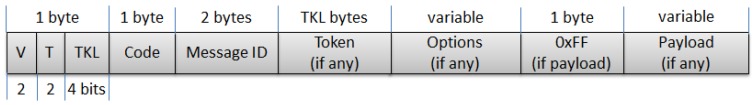
CoAP Message Format consisting of a 4-bytes base binary header followed by optional extensions.

CoAP uses the same RESTful principles as HTTP, but it is much lighter so that it can run on constrained devices [6,7]. To achieve this, CoAP has a much lower header overhead and parsing complexity than HTTP. It uses a 4-bytes base binary header that may be followed by compact binary options and payload. Figure 1 shows the CoAP message format as specified in version 18 of the draft. This version was approved by the Internet Engineering Steering Group (IESG) in July 2013 and was at the time of writing this article being edited by the RFC editor to convert the draft into an RFC. Thus, it is expected that this will be the final CoAP message format.

The CoAP interaction model is similar to the client/server model of HTTP. A client can send a CoAP request, requesting an action specified by a method code (GET, PUT, POST or DELETE) on a resource (identified by a URI) on a server. The CoAP server processes the request and sends back a response containing a response code and payload. Unlike HTTP, CoAP deals with these interchanges asynchronously over a datagram-oriented transport layer such as UDP and thus also supports multicast requests. This allows CoAP to be used for point-to-multipoint interactions which are commonly required in automation. Optional reliability is supported within CoAP itself by using a simple stop-and-wait reliability mechanism upon request. Secure communication is also supported through the optional use of Datagram Transport Layer Security (DTLS) [8]. As can be seen in Figure 1 all CoAP messages start with a 4-bytes base binary header that consists of the following fields:

- *Version (V)*: indicates the CoAP version number. Current version is 1.
- *Type (T)*: indicates if this message is of type Confirmable, Non-Confirmable, Acknowledgement or Reset.
- *Token Length (TKL)*: indicates the length of the variable-length Token field.
- *Code*: indicates if the message carries a request (1–31), a response (64–191), or is empty (0). In case of a request, the Code field indicates the Request Method (GET, POST, PUT and DELETE); in case of a response a Response Code.
- *Message ID*: is used for the detection of message duplication, and to match messages of type Acknowledgement/Reset to messages of type Confirmable/Non-confirmable.

To be able to offer communication needs that cannot be satisfied by the base binary header alone, the base 4-bytes header may be followed by one or more of the following optional fields:

- *Token*: the Token is used to correlate requests and responses.
- *Options*: an Option can be followed by the end of the message, by another Option, or by the Payload Marker and the payload.
- *Payload*: if present and of non-zero length, it is prefixed by a fixed, one-byte Payload Marker (0xFF) which indicates the end of options and the start of the payload. The payload data extends from after the marker to the end of the UDP datagram, *i.e.*, the Payload Length is calculated from the datagram size. The absence of the Payload Marker denotes a zero-length payload.

CoAP defines a number of options which can be included in a message. Both requests and responses may include a list of one or more options. Each option instance in a message specifies the Option Number, the Option Length and the Option Value of the defined CoAP option. As an example of a simple CoAP option consider the Content-Format option. This option indicates the representation format of the message payload. This option has the Option Number 12 and its Option Length is between zero and two bytes. The Option Value itself is a numeric content format identifier that is defined in the CoAP Content Format Registry (Section 12.3 of the draft [5]). Another example is the Max-Age option which has the Option Number 14. This option indicates the maximum time a response may be cached before it is considered not fresh. The Option Value is an integer number of seconds between 0 and 2^32^−1 inclusive (about 136 years). If this option is not included in any CoAP response, it can be assumed that the response will be fresh for 60 s and thus will not be queried again by a cache within this time frame.

When using confirmable messages CoAP tries to achieve reliability by using a simple stop-and-wait retransmission with exponential back-off. By default the initial back-off is set to a random time between 2 and 3 s. This means that if a reply to a confirmable packet is not received within the initial back-off time, the CoAP sender will double the initial back-off time and retransmit the packet. If a reply to the first retransmission is not received, CoAP will again double the back-off time and retry the transmission until MAX_RETRANSMIT (by default 4) is reached. If no reply is received after expiring of the back-off time of the last retransmission, the client will be notified about the error condition.

The IETF CoRE working group considers constrained RESTful environments as an extension of the current web architecture. The group envisions that CoAP will complement HTTP and that CoAP will be used not only between constrained devices and between servers and devices in the constrained environment, but also between servers and devices across the Internet [9]. An important requirement of the CoRE working group is to ensure a simple mapping between HTTP and CoAP so that the protocols can be proxied transparently. Thus proxies and/or gateways play a central role in the constrained environments architecture. These proxies have to be able to communicate between the Internet protocol stack and the constrained environments protocol stack and to translate between them as needed.

### 2.2. Resource Discovery

**Figure 2.**
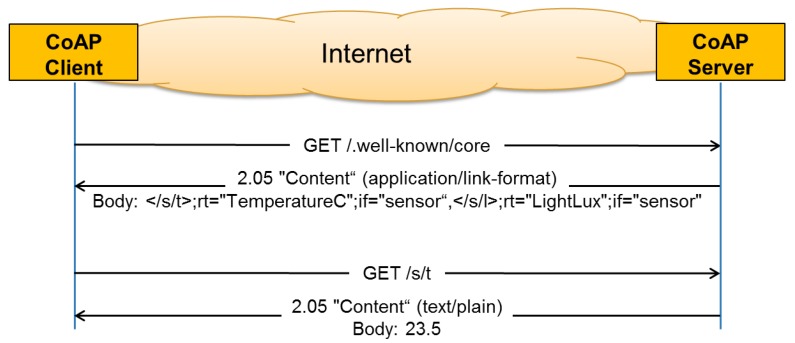
An example of Constrained RESTful Environments (CoRE) direct resource discovery and Constrained Application Protocol (CoAP) request.

In machine-to-machine (M2M) applications where there are no humans in the loop, it is important to provide a way to discover resources offered by a constrained server. For HTTP Web Servers, the discovery of resources is typically called Web Linking [10]. The use of Web Linking for the description and discovery of resources hosted by constrained web servers (CoAP or HTTP) is specified by the CoRE Link Format- RFC 6690 [11]. A well-known relative URI “/.well-known/core” is defined as a default entry-point for requesting a list of links to resources hosted by a server. Once the list of available resources is obtained from the server, the client can send further requests to obtain the value of a certain resource. The example in Figure 2 shows a client requesting the list of the available resources on the server (GET /.well-known/core). The returned list (in CoRE Link Format) shows that the server has, amongst others, a resource called /s/t that, when queried, returns the temperature in degrees Celsius. The client then requests the value of this resource (GET /s/t) and receives a plain text reply from the server with the value of the current temperature as payload of the message (23.5).

**Figure 3.**
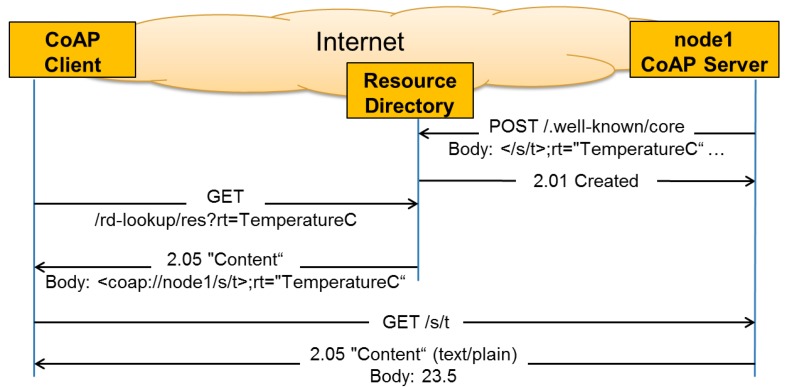
An example resource discovery by using a Resource Directory.

However in many M2M scenarios, nodes might have long sleeping periods and thus making direct discovery of resources not practical. To solve this problem, the CoAP community is proposing to use CoRE Resource Directories (RD) that host descriptions of resources held on other servers [12]. This way a CoAP server can register its resources with one or more RDs. Clients in turn can discover these resources by performing lookups against an RD. For example the same resource discovery that was performed by using direct communication between the client and the server in Figure 2 can now be performed by using an RD as illustrated in Figure 3. For more details about the registration and lookup interfaces of Resource Directories we refer to [12].

### 2.3. Blockwise Transfer

In many cases the payloads that CoAP needs to carry are very small (e.g., just a few bytes for temperature sensor, door lock status or toggling a light switch). In these cases the basic CoAP message provides very efficient means of communication and works very well. However in some cases CoAP needs to handle larger payloads (e.g., images or firmware update). Since CoAP is based on datagram transports such as UDP or DTLS, data fragmentation and reassembly is not offered by these transport protocols. Relying on IP fragmentation is also not very helpful, because IP fragmentation can handle only payloads up to 64 KB. Thus, providing a mechanism at the application layer that is able of transferring large amounts of data in smaller pieces becomes a necessity. This will not just help avoiding the 64 KB UDP datagram limit, but will also help avoiding both IP fragmentation (MTU of 1280 for IPv6) and also 6LoWPAN adaptation layer fragmentation in LLNs (60–80 bytes).

To overcome the payload size limitation, draft-ietf-core-block defines two CoAP options: Block1 and Block2 [13]. By using this pair of options CoAP becomes capable of transferring a large payload in multiple smaller CoAP messages. Both Block1 and Block2 options can be present both in request and response messages. In either case, the Block1 Option pertains to the request payload, and the Block2 Option pertains to the response payload. Block sizes are represented inside the Block1 and Block2 Options as a three-bit unsigned integer called *SZX* indicating the size of a block to the power of two. Thus:

(1) $$\text{blocksize}=2^{\text{SZX}+4}$$

The allowed values of *SZX* are 0 to 6 and thus the resulting allowed block sizes are: 16, 32, 64, 128, 256, 512 and 1024 bytes.

6LoWPAN might start using fragmentation/reassembly for datagrams as soon as the payload size gets larger than 60 bytes. This fragmentation/reassembly process burdens the lower layers with conversation state and is sometimes not implemented to conserve resources at the constrained devices. To avoid such fragmentation and reassembly, blockwise transfer with Block1 and Block2 sizes of 16 or 32 should be used whenever the payload exceeds 60 bytes.

An important aspect of the blockwise transfer mechanism is that often the server can handle block transfers in a stateless fashion. It does not require connection setup and the server does not need to track each transfer separately and thus conserves memory.

### 2.4. Group Communication

The IETF CoRE working group has recognized the need to support a non-reliable multicast message to be sent to a group of devices to manipulate a resource on all the devices in the group. Therefore, they have developed the “Group Communication for CoAP” Internet Draft [14], which provides guidance for how the CoAP protocol should be used in a group communication context. *Group Communication* refers to sending a single CoAP message to all members of a specific group by utilizing UDP/IP multicast for the requests, and unicast UDP/IP for the responses (if any). This implies that all the group members (the destination nodes) receive the exact same message. The solution proposed by the IETF CoRE working group is discussed further in Section 4.

## 3. Group Communication Requirements

In our work we broaden the *CoAP group communication* definition from Section 2.4: CoAP-based group communication is a *method to manipulate a group of resources on devices using CoAP as the underlying protocol*. Such a group of resources is called an *entity* and the resources themselves are called the entity *members*. We classify two types of entities based on the entity members:

- *Homogeneous Entity*: is an entity in which the members share a common set of properties (URI path, method, content-type, block-size, observe, *etc*).
- *Heterogeneous Entity*: is an entity in which not all members share a common set of properties.

Within this context, we now define the requirements and goals for CoAP group communication and motivate their importance in the context of IoT applications, constrained devices and LLNs:

(1) *Flexibility*: as it is expected that the IoT will contain a huge amount of devices, it is also expected that the amount of device types will be enormous. To interact with a subset of these devices in a group, the group communication solution should be very flexible to accommodate the differences between devices and device types. In particular the solutions should be flexible enough to offer:
  a. Support for homogeneous and heterogeneous groups. CoAP servers may be heterogeneous in terms of their CoAP resources, even if they provide the same functionality. For example the IPSO (Internet Protocol for Smart Objects) Alliance has published an Application Framework that recommends a classification of resources based on their functionality by defining a set of Resource Types [15]. However even if a group of resources offers the same functionality (same resource type), the actual resource path of the resource on different group members might be different. In some cases one might even want to have a group of resources with different resource types and simply query it (e.g., collecting heterogeneous environmental data). Also other types of heterogeneity in the resources are possible: e.g., different payload to PUT request, different media-types, *etc*.
  b. Support of group members that are not part of the same network. Often, it is assumed that all members in a group belong to the same network. However, group communication solutions should not be limited to this setting. In the future, it may as well be that group communication involves nodes from different sensor networks, networks that may be co-located or spread over different locations.
(2) *Light-Weight (footprint)*: the group communication solution should have limited footprint on constrained devices. It is expected that a lot of the IoT devices will be of Class 1 (∼10 KB of RAM, and ∼100 KB of ROM) [16]. Any overhead involved by a group communication solution should not prevent the solution from running on Class 1 devices. Furthermore the solution should scale with the number of groups a certain member can be part of.
(3) *Use of Standards*: to allow the creation of groups across a variety of members from different vendors and domains, it is mandatory to use standard protocols that are widely supported. The focus of our work is on using CoAP as an application layer standard protocol. As mentioned in Section 2, CoAP consists of a base protocol and a set of optional extensions. It is expected that not all CoAP servers will support all CoAP extensions. Thus it becomes essential to limit the use of optional extensions in order not to exclude potential CoAP servers of becoming group members due to missing extensions.
(4) *Performance*: CoAP is designed to run on resource constrained devices. In order to keep it this way, any CoAP group communication solution should have little overhead and be efficient in the use of resources of the nodes and the LLN. In particular the number and size of messages sent in the LLN should be kept to a minimum, in order to conserve valuable node energy (nodes are often battery powered, or harvest energy from the environment). A very powerful method for limiting the number of messages inside the LLN is using efficient caching techniques. This not only limits the number of messages and energy consumption, but it also decreases response latency. CoAP transactions and options are thus well optimized to support caching whenever possible. Group communication should not be an exception and should not hinder the use of caches.
(5) *Validation and Error Handling*: since a group might include heterogeneous members, it should be possible to validate the group in order to make sure that the group works as intended. The group should have mechanisms for reporting and handling error conditions such as node or route failures.
(6) *Reliability*: sometimes it is not essential to get reliable replies from all group members (e.g., it might be enough to get the temperature measurements from just one of the many temperature sensors in a room). However in many other cases, it can be important to have reliable communication with all group members. For example, one would expect that all lights in the room would go on when one flips on the light switch.
(7) *Ease of Group Manipulation*: the needs of the user might change with time and thus group membership might also change. In dynamic environments the changes might be frequent. It is important to be able to handle such changes easily. One should avoid node reconfigurations, as this might be a tedious task. Also it should be possible for nodes to be part of different groups at the same time or at different times.
(8) *Expressiveness/Control*: there are several results that one might want to achieve by interacting with a group of objects/object resources. In some cases one might be interested in all the individual results of all members as in the case of turning the lights on. In many other cases the individual values might not be of interest at all. In these cases one might be interested in an aggregated value (e.g., min, max, avg,…) of all, or even of just a subset of, the group members. Thus it is desired to have support for processing of individual group member results and replying to the requester with aggregated results. For example it should be possible to query a certain subset of the members and compute the average, or reliably update all members.
(9) *Security*: secure communication might be of little interest inside a shielded and controlled environment. However, by exposing sensors and actuators to the Internet, security becomes a major concern. In some scenarios having an end-to-end security is a strict requirement. Communicating with a group of resources is no exception. In fact it is even more sensitive than communicating with an individual resource, since compromising the group means compromising all the individual members.

## 4. Existing Solutions

As mentioned, to address the group communication needs, the IETF CoRE Working Group has developed the “Group Communication for CoAP” Internet Draft [14]. This draft discusses fundamentals and use cases for group communication patterns with CoAP and provides guidance for how the CoAP protocol should be used in a group communication context. The draft provides an approach for using CoAP on top of non-reliable IP multicast and does not attempt to provide a reliable solution for CoAP group communication as set forward by the Working Group charter. Certainly the use of multicasts allows reducing the amount of requests in the LLN, by sending one request to several destinations at the same time. However, multicasts are not cache-friendly, preventing possible reduction of requests and replies by utilizing caches. Depending on the use case and network topology, the reduction of packets as a result of using a cache can be better than the reduction obtained from using multicasts. This approach exhibits the limitations of multicasts as discussed in Section 1. Also multicasts are not useful when a single user action needs to trigger different sensor requests, since one multicast request delivers the same message to all group members. Additionally, secure communication with the group members is not possible, since all communication based on this draft operates in CoAP NoSec (No Security) mode. Finally, multicast is not supported on all LLN MAC protocols, especially MAC protocols that use Radio Duty Cycles (RDC) to shut down their radios when not in use. For example Xmac does not support multicast since it shuts down its receiver to avoid overhearing. Special MAC protocols that support multicast have been proposed such as in [17]. Interestingly, this MAC protocol will send the multicast data to each receiver one by one (unicast) if the multicast data drops below a certain threshold.

As mentioned, the use of multicast as a means to interact with multiple objects concurrently requires multicast support in the network. Typically IP multicast relies on topology maintenance mechanisms to discover and maintain routes to all subscribers of a multicast group. However, maintaining such topologies in LLNs may not be feasible given the available resources. As a result, special multicast protocols have been proposed for the use inside LLNs. For example, the “Multicast Protocol for Low power and Lossy Networks (MPL)” internet draft uses the Trickle algorithm to manage message transmissions for both control and data-plane messages and avoids the need to construct or maintain any multicast forwarding topology [18]. An alternative is the stateless multicast RPL forwarding algorithm (SMRF), which according to [19] achieves significant delay and energy efficiency improvements at the cost of a small increase in packet loss. Regardless of the used multicast protocol, all nodes on the path between the sender and receivers must be extended to support the protocol.

The “Group Communication for CoAP” Internet Draft was the basis for the work presented in [20], in which web services based CoAP multicasts were used to access data from Building Automation Systems (BAS). It shows how using multicasts allows creating basic building control scenarios without the need of a central control unit. Certainly this approach has several advantages such as eliminating the need for a control unit, often less power consumption than using unicasts and its suitability in many non-critical use cases (due to the lack of reliability of multicasts). However this approach exhibits the limitations of multicasts as discussed in this section above.

Simple unicast solutions are defined in the CoRE Interfaces draft [21]. Among other interface types, this draft defines the Batch interface type and its extension, the Linked Batch interface type. Batch interfaces are used to manipulate a collection of sub-resources at the same time. Contrary to the basic Batch, which is a collection statically defined by the web server, a Linked Batch is dynamically controlled by a web client. The resources forming the linked batch are referenced using Web Linking [10] and the CoRE Link Format [11]. The draft does not foresee any way to manipulate resources that are located on multiple smart objects with a single client request.

An approach somewhat more similar to ours, also using the notion of an entity, has been presented in [22]. The aim here is to annotate real-world objects by using entities that are automatically created based on semantic information, which resides on the constrained devices. One problem of using semantics on constrained devices is that semantics can easily require a lot of memory that might not be available on the constrained devices. Further, in our approach users can create entities as required and we address important aspects related to entity validation and entity behavior.

The authors of [23] present an extension to CoAP called SeaHttp, that enables communication with a group of resources. Similar to our work, SeaHttp also uses unicasts to realize group communication. The authors propose to extend CoAP with two additional methods (BRANCH and COMBINE) to allow members to join and leave groups without the need for a separate group manager. This means that members should have the intelligence to know which group they should join/leave. Constrained devices will not have this intelligence, so again, a “manager” will be needed to inform the devices so they can take appropriate actions. Furthermore, BRANCH and COMBINE can maybe reduce the number of messages; however the trade-off is the need to implement a new mechanism. It is better to use an approach that can be plugged in into any existing network without major modifications (or at least not a modification to every node). The article does not discuss if the use of caches will still be possible with SeaHttp resources. However, should this be possible then also the caches should be extended accordingly. Finally this approach does not have the flexibility we target, since group members have to be reprogrammed with the groups they should join each time the requirements of the user changes.

To our knowledge, these are the only works that explore communication solutions for interacting with a group of CoAP-enabled constrained devices. Next to these, there exist other solutions to realize or improve multicast communication in Wireless Sensor Networks, such as [18,24]. These solutions can alleviate some of the problems related to (reliable) multicasting, but their scope is different from the work presented here.

## 5. Group Communication Using Unicasts

We aim to create an intermediate level of aggregation to be able to easily manipulate a group of resources across multiple smart objects. To avoid increasing the *footprint* of the constrained devices, we use the *same technology* as used to manipulate individual resources, *i.e*., CoAP, and extend it accordingly. Such a group of resources is called an *entity* and the entity can be used or manipulated through a single CoAP request. Similarly, the creation of an entity by a client is realized via a single CoAP request and includes a complete *validation* of the entity. Furthermore we introduce the notion of profiles for the created entities. The use of entity profiles allows the client to specify in more detail how the *entity should behave* (e.g., if it should use confirmable or non-confirmable CoAP messages), and, through updating the profile, allows manipulation of this behavior. As such, we strive to combine ease of creation, ease of usage and *flexibility* in behavior into a complete solution for interacting with CoAP resources from different objects inside a LLN. By building upon *standardized* concepts, the impact on the constrained devices is limited. In the following subsections we discuss the details of our approach.

### 5.1. System Overview

**Figure 4.**
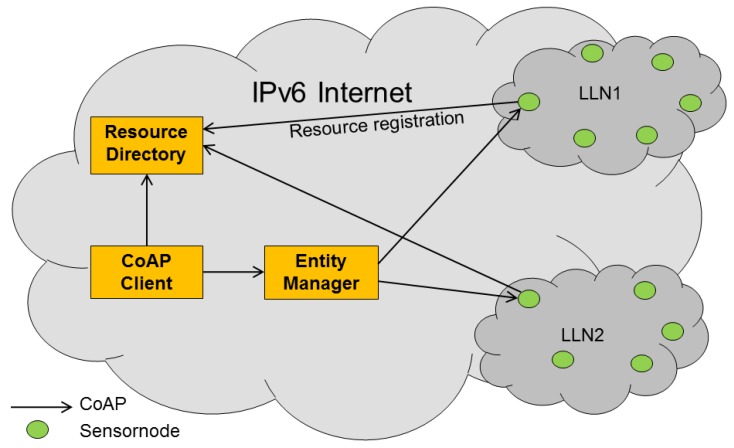
Clients create entities consisting of several smart object resources on the Entity Manager. Clients can optionally query a resource directory to discover the existence of the resources.

We call the component that manages the entities, the Entity Manager (EM). This component, which can reside, e.g., on the Border Gateway of the LLN, is responsible for maintaining entities that are created from groups of resources residing on CoAP servers (*i.e.*, sensors and actuators) inside the LLN. Clients on the Internet can interact with an EM to create new entities and/or customize how these entities should behave. Optionally the client can elect to contact a resource directory [12] in order to discover which resources are available in the network. Figure 4 shows an overview of the involved components.

**Figure 5.**
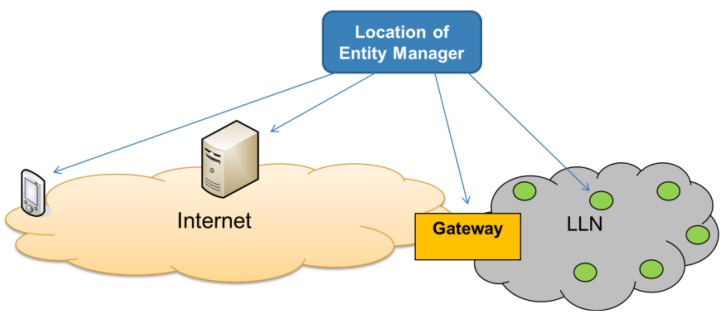
The Entity Manager functionality can be integrated into any CoAP server. The optimal location for the EM depends on the use case.

The EM functionality does not have to be put on a dedicated device. Theoretically any CoAP server can be extended to become an EM (Figure 5). The choice of the most appropriate location to put the EM functionality depends on the size and topology of the network. For example, it can reside on a smart object in the constrained network with enough resources, in the Cloud, on the client device itself, or on a gateway at the edge of the LLN. The latter case has the added benefit that security can be centrally managed besides offloading the processing from constrained devices. One can also decide to implement multiple EMs (at the same or at different locations) to avoid having a single point of failure and thus improving reliability, availability and scalability.

Regardless of the location of the EM, it will serve as a “proxy” between the client and the constrained devices. Client requests will be sent to the EM, which will analyze and verify the requests and then issue the appropriate requests to the constrained devices using CoAP. Once the EM receives responses from the constrained devices, it will combine them according to the needs of the client and will send back an aggregated response to the client.

**Figure 6.**
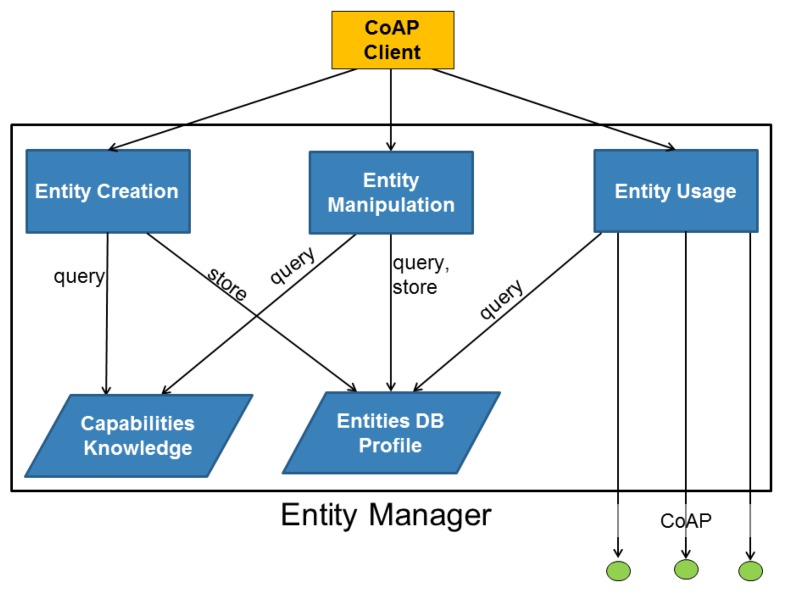
Entity Manager (EM) high-level structure.

When a client tries to create a new entity consisting of a group of resources inside LLNs, the EM performs a sanity check on the request in order to make sure that the resulting entity would make sense. For example it verifies that the resources inside the entity are valid, whether they support a certain content format and whether their data can be aggregated. Customization of the entity behavior is accomplished by creating profiles for the entities. A profile of an entity can specify for example whether to return the values of all resources in the entity, only the computed average of all values or a subset of all values. Figure 6 shows a high-level structure of the Entity Manager. It shows that the EM contains two databases:

- *Entity Database*: in this database all entities are stored along with their profiles as defined by the user.
- *Capabilities Database*: this optional database provides rules and knowledge that can be used to match user requests with sensor capabilities. This can be as simple as translating a request for temperature in degrees Celsius while obtaining the data from a sensor that only supports Fahrenheit. It can also be more complex, e.g., converting resource representations from one content format into the other.

### 5.2. Entity Creation

To facilitate the creation and manipulation of entities, the Entity Manager offers a CoAP resource “/e”. We call this resource the Entity Management Resource. This interface only supports the CoAP POST request method. As payload of the request, it expects a collection of resources in CoRE link format [11], which together should form the entity. In the response, the Location-Path CoAP option is used to specify the name of the newly created resource. In the current design, the payload of the response is in plain text and describes the results of the validation tests performed by the Entity Manager on the collection of resources.

Thus, when a client wants to create an entity consisting of several members, it has to compose a CoAP POST request and send it to the Entity Management resource on the Entity Manager. The EM creates the entity, assigns it a unique URI, and stores the entity in the entity database for future usage. Then the EM starts the entity validation process (explained in the next subsection). The client is informed about the URI to use in order to access or further customize the newly created entity and about the results of the validation of the entity. In addition, the new entity resource can be registered in a resource directory as well, making it available for lookup. If the entity did not pass the validation process the client should fix any errors and resubmit the entity for validation again before the client can use the entity.

**Figure 7.**
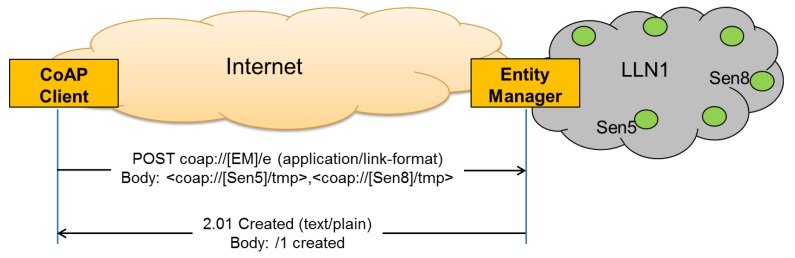
A CoAP client requesting from an Entity Manager (EM) to create a new entity that contains two resources.

An example of the entity creation process is shown in Figure 7. In this simple example the client requests the creation of an entity consisting of two members: coap://[Sen5]/tmp and coap://[Sen8]/tmp, with Sen5 and Sen8 the IPv6 addresses of the two sensors. The Entity Manager creates the new entity, assigns it the URI “/1” and informs the client about the newly created entity. From now on, any client can access the newly created entity by accessing the “/1” resource on the EM. Please note the validation process is not shown in Figure 7 for simplicity.

At creation time, the client can use optional URI-Query CoAP options with the POST request to specify the name of the entity to be created or to customize the default behavior of the entity. For example, a POST to coap://[EM]/e?path= “/room_humidity”&eo= “min” will create the entity “room_humidity” that returns by default the minimum value of all members when queried. We will discuss customization of the entity behavior in more detail in Section 5.5.

### 5.3. Validation Process

Whenever a client requests to create a new entity or to modify an existing entity, the EM performs a validation process. The purpose of this validation process is twofold:

(1) Make sure that the members in the entity exist and can be used.
(2) Derive the properties of the entity based on the properties of the members it contains.

If the entity successfully passes validation the EM marks the entity as a valid entity and stores the entity along with its calculated properties in the entity database for future usage. If the entity fails validation it is still created, but marked as invalid. The entity validation is based on EM's knowledge of the individual members and their profiles and based on the knowledge in the capabilities database as will be discussed in the next paragraphs.

Resource profiles can be used to express capabilities of a CoAP server and its resources [25]. Profiles are usually expressed in JSON format [26]. To briefly illustrate resource profiles, let us assume that in Figure 7 the temperature sensor at “coap://[sen5]/tmp” supports the “Uri-Host” (3), “ETag” (4), “Observe” (6), “Uri-Port” (7), “Uri-Path” (11) and “Content-Format” (12) CoAP options (op). This sensor further supports the “application/json” (50) content format (cf) and the allowed method (m) is GET (1). This will result in sen5 having the following profile:
Res: 2.05 Content (application/json) {  “profile”: [   {    “path”:”temp”,    “op”:[3,4,6,7,11,12],    “cf”:[50],    “m”:[1]   }  ] }

If the Entity Manager does not know any of the members in an entity (e.g., based on knowledge in a resource directory) or does not know the member capabilities, it tries to obtain this information according to a fallback mechanism as follows:

(1) The EM tries to contact the object containing the resource in order to obtain the resource profile, since this returns the most complete information about the resource.
(2) If the resource profile does not exist, the EM tries to derive information about this resource from /.well-known/core of the respective object.
(3) If this fails as well, the EM tries to query the resource directly to discover, as a minimum, if the resource exists or not.

**Figure 8.**
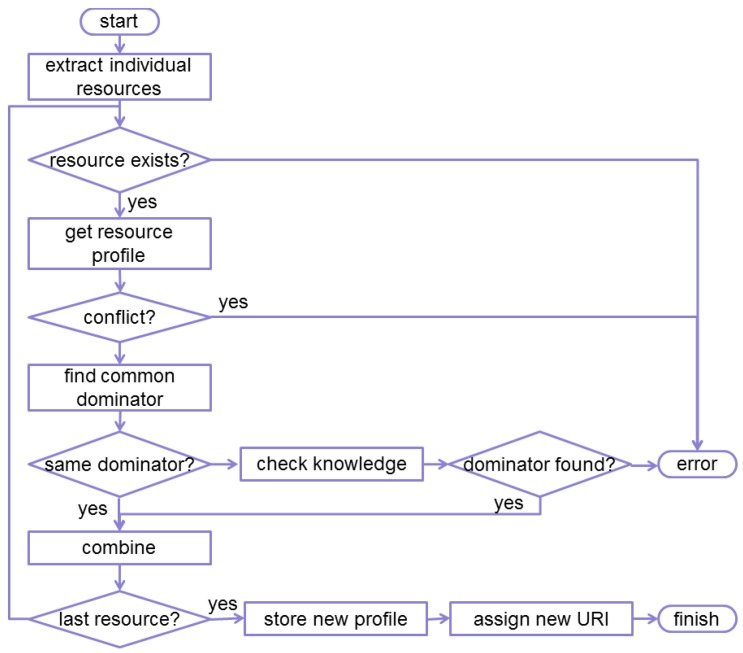
Entity validation process flow.

The validation process that the Entity Manager performs on entities is shown in a simplified form in Figure 8. In essence the process will:

- Verify whether the individual members contained in the entity are valid (*i.e*., the resources exist on the respective nodes).
- Derive the operations that can be performed on the entity, based on the operations supported by the individual members (e.g., which CoAP options are supported, which RESTful methods are allowed?).
- Verify whether the individual members do not conflict. A sample conflict can occur when an entity creation request contains a sensor member that supports only the GET method and an actuator member that supports only the PUT method.
- Verify whether the responses sent by the individual members can be combined together using a common denominator or knowledge from the capabilities database.

Once the EM knows all information about the members that should become part of the entity and once all necessary checks have been passed, the EM creates a profile for the entity based on this information and the EM's capabilities database. To illustrate this let us further assume that the second temperature sensor in Figure 7 “coap://[sen8]/tmp” supports the same options as sen5 except for the observe option. Only the GET method is allowed and the supported content formats on this sensor are “text/plain” (0) and “application/json” (50). Thus sen8 will have the following profile:
Res: 2.05 Content (application/json) {  “profile”: [   {    “path”:”tmp”,    “op”:[3,4,7,11,12],    “cf”:[0,50],    “m”:[1]   }  ] }

Based on these two profiles the EM constructs a profile for the newly created entity. This profile contains information related to the resource itself, as described in [25]. In this example, this includes the options that are supported, the supported methods (only GET) and the content format “application/json” (50). In addition, the profile is extended with an entity specific part, providing more information about the entity itself. The resulting profile of the entity looks as follows:
Res: 2.05 Content (application/json) {  “profile”: [   {    “path”:”1”,    “op”:[3,4,7,11,12],    “cf”:[50],    “m”:[1]   }  ],  “entity”:[   {    “er”:”coap://[sen5]/tmp,coap://[sen8]/tmp”,    “eo”: [“ lst”, “avg”, “min”, “max”]   }  ] }

This simple example illustrates how an entity profile is constructed; either based on information from individual resource profiles or based on information retrieved via other means such as resources attributes derived from /.well-known/core. Much more information than shown here can be included and, by using a flexible representation format, the profile concept can be easily extended with new information.

The entity specific part of the profile currently supports the following fields:

- *Entity Resources (er)*: a list of the individual resources out of which the entity is composed.
- *Entity Message Type (emt)*: specifies the message type to be used for communication between EM and members. Possible values are *con* (confirmable) and *non* (non-confirmable). The default is *con*.
- *Entity Number of Replies (enr)*: specifies the number of replies that should be received from the members before sending a reply to the client. This makes it possible not to wait for all members to reply. The default behavior is to wait until all replies have been received or have timed out.
- *Entity Operation (eo)*: The operations that can be performed on the results obtained from the members. The operation is used to combine replies received from all the members (or the number of replies specified by enr) into one reply to the client. If at the time of querying the entity the client does not specify which operation to use, the first operation listed in this field will be used. Currently the following Entity Operations are supported:
  ○ *List (lst)*: A list of replies received from the members, without any arithmetic processing. This is the default behavior if no entity operation was specified.
  ○ *Average (avg)*: The average value.
  ○ *Minimum (min)*: The minimum value.
  ○ *Maximum (max)*: The maximum value.
- *Delay between Requests (delay)*: specifies the delay that should be injected between the requests sent from the EM to the members. The default is 0 and thus the EM will send the requests as fast as it can.

These entity profile fields can be provided by the client upon creation time. If no values are provided, the EM will use default values for the newly created entity. To construct the entity profile, the EM uses its internal knowledge to offer additional features that are not provided by the individual members. For example, the EM can interpret certain member payloads, convert between content formats and return the entity result in particular content format. Currently we support conversion between plain-text, JSON and RDFN3 content formats for numerical sensor values. The list of conversion functions can be extended easily.

### 5.4. Entity Usage

**Figure 9.**
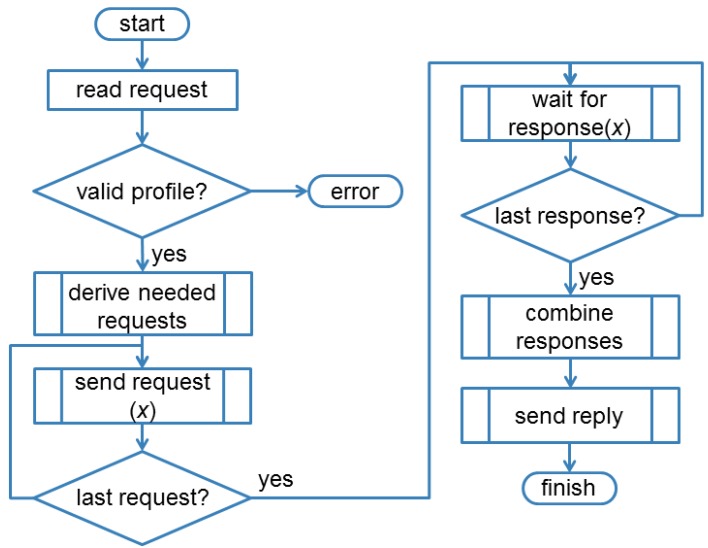
Simplified entity usage process flow.

Once an entity has been created, a response is sent back to the client. This response contains the URI of the entity, which was either requested by the client or assigned dynamically by the EM. The client can now interact with the entity by issuing a single CoAP request to the resource representing the entity. When a request for an entity arrives, the process flow shown in Figure 9 is executed. The EM breaks down the request into its components and sends the individual requests to the respective smart objects using unicast CoAP messages. It can either do that in parallel or sequentially with a configurable delay between requests to the members in order to avoid potential network congestion. Once all needed answers have been received, the EM creates a response to the client based on the individual responses and sends it to the client. Note that aspects such as how many members should respond, how the response is composed, how it should look like, *etc*. depend on the entity profile and can be customized using URI queries as will be explained later on.

**Figure 10.**
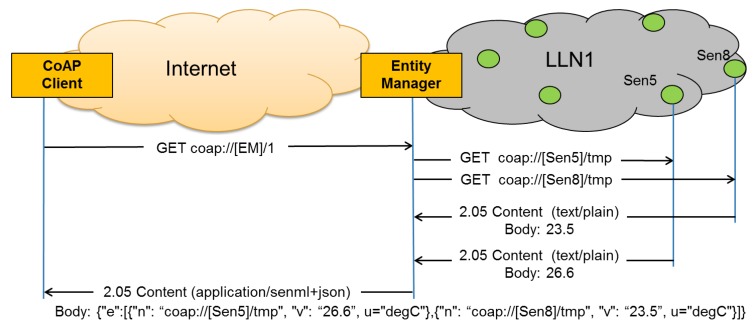
A CoAP client requesting from an Entity Manager to obtain the values for the entity that was previously created in Figure 7.

Figure 10 shows an example of using the entity that was created previously in Figure 7. The client issues a GET request on the entity's resource “/1”. This results in the EM issuing two GET requests to the individual members, waiting for replies from both of them and then sending both results in one combined response back to the client.

The client can decide to query the entity using its default behavior as described in the entity profile or to customize its behavior. To customize the behavior the client can include URI queries in its request to the entity. The supported URI queries that can currently be used are: Entity Operation (eo), Entity Number of Replies (enr) and Delay Between Requests (delay) as described in Section 5.3. For example, to obtain the average value of the two temperatures of the entity /1 in Figure 10, the client should use the URI: coap://[EM]/1?eo=”avg” and should use coap://[EM]/1?enr=”1” to indicate to the EM that it is enough to send just any one of the two member replies as a reply to the entity. This last example demonstrates how our solution can be used to achieve a behavior similar to anycast requests when there are redundant members available.

### 5.5. Entity Modification and Behavior Manipulation

It is possible that a client wants to modify an entity after its creation. For example, a client might want to add new members to the collection of members in the entity or remove a number of members. Alternatively, the client may want to customize the behavior of an existing entity. The latter can include aspects such as the default number or percentage of members that should respond before the entity manager replies to the client, the default content format of the response, the default operation (e.g., average, max, min, *etc*.) that should be performed on the results before sending them to the client, *etc*. Modifications to the entity or to its behavior can be made by updating the entity's profile and posting the updated information (PUT or POST) to coap://[EM]/.well-known/profile?path=”[ENTITY_URI]”, in which /.well-known/profile is a resource for accessing the profile of a resource as described in [25] and ENTITY_URI the URI of the entity, e.g., “/1” in our example. When a client wants to modify the profile of an entity, this information is passed to the EM, which will validate the request and change the profile if the validation was successful. Finally, removing an entity can be realized by sending a GET request to the entity management resource that includes action=”delete” URI query and specifying the entity to be deleted, e.g., coap://[EM]/e?path= “ENTITY_URI”&action= “delete”.

## 6. Implementation and Evaluation

Our solution described above enables the use of unicast messages as an alternative to using multicasts for realizing CoAP group communication. In order to evaluate our solution and to show how it can be used in a real-world scenario we have implemented it and built a demo box for demonstration purposes. In this section we present the implementation of our solution and a basic description of the demo box followed by functional and performance evaluation.

### 6.1. Implementation

The key in our group communication solution is the Entity Manager. We have implemented the Entity Manager functionality on the gateway of the LLN using the CoAP++ framework [27]. The framework itself and the Entity Manager implementation on top of it have been realized in Click Router, a C++ based modular framework that can be used to realize any network packet processing functionality [28]. The CoAP++ implementation on the gateway also includes a resource directory and a cache that are used in the evaluation tests.

As group members we have used Zolertia Z1's boards [29] that run the popular Contiki 2.6 operating system [30]. This version of Contiki was the current version when we started our experiments and included a stable implementation of CoAP, namely the Erbium CoAP server [31]. Our group communication approach does not require any changes on the CoAP enabled constrained devices. However, in order to demonstrate how the EM can use resource profiles to validate entities, we have added resource profiles to the constrained CoAP servers. Additionally, in order to be able to compare the performance of our solution with a multicast based solution, we have added multicast support to Contiki by using an open source implementation [32].

The CoAP++ framework's interoperability with the Erbium CoAP server as well with other CoAP implementations has been formally tested by the European Telecommunications Standards Institute (ETSI), a non-profit standards organization, in three events called CoAP Plugtests [33].

**Figure 11.**
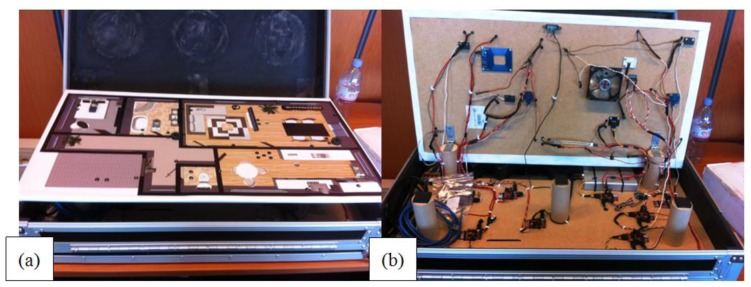
iMinds IoT portable home automation demo box. (**a**) The upper level shows a map of a house with various sensors and actuators installed; (**b**) Looking at the lower layer of the box the connections of the sensors are shown.

To demonstrate the practical use of our solution we have built a portable demo box (Figure 11). The box has two layers. The top layer has a floor plan of a house with several rooms. Each of the rooms is equipped with some wireless sensors and some wireless actuators. The top layer can be easily tilted to reveal the wireless sensor network that consists of eight wireless sensor nodes that are mounted on the back of the top layer and the bottom layer. The wireless sensor nodes are in the form of Zolertia Z1's boards that are running the Contiki operating system. Each of these nodes has been equipped with a number of sensors and actuators. The sensors include light intensity, temperature, proximity, movement (PIR), force, RFID and magnetic switch sensors. Supported actuators include multiple lights (in the form of LEDs) and a cooling fan. These sensors and actuators each have a corresponding CoAP resource. One of the wireless sensor nodes runs a 6LoWPAN border router and is connected to an Internet gateway. The gateway for this network is an Alix system board running voyage Linux. Apart from routing traffic, the gateway also provides the Entity Manager services.

Using this demo box we are able to show several home automation scenarios that use our group communication solution. For example it is possible to turn on all lights in a room (or a set of rooms) when the pressure sensor in the bed indicates that the person has left the bed while it is dark in the room. For more details about our demo box we refer to [34].

Besides its function as a showcase for our CoAP implementations, we have used the demo box for the functional evaluation of our group communication solution. However the demo box does not provide a suitable environment for good performance evaluation for several reasons. Since the box is relatively small, all radios are very near to each other and build a full-mesh single hop topology. This makes it impossible to perform multi-hop experiments. Furthermore, since the number of nodes in the demo box is only eight nodes, no larger scale experiments can be performed. Consequently, in order to obtain good insights in the proposed solution and to obtain a comprehensive performance evaluation it is needed to turn to either a simulation environment or larger scale testbeds. For this paper, we opted for the first, *i.e.*, a simulation study using the Cooja network simulator, which is part of the Contiki operating system. The simulation environment allows both a functional and performance evaluation, with the demo box complementing the functional evaluation.

The simulation environment enables the initial evaluation of the performance of our solution for varying entity sizes and number of hops to the entity resources. Evaluation on larger real-life testbeds will prove useful for validating the simulation experiments and for conducting experiments in more dense and more realistic (e.g., Wi-Fi interference) environments. However this will take a significant amount of time and is beyond the scope of this work.

### 6.2. Functional Evaluation

**Figure 12.**
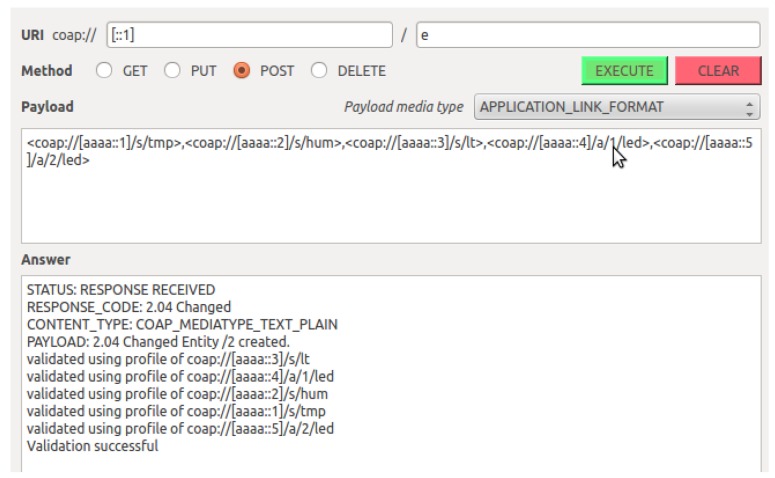
Sending a CoAP POST request to the Entity Manager to create an entity with five members, results in the creation of the entity with the URI “/2” and in the validation of the entity.

The functionality for creating, validating, using and deleting entities has been implemented as described above. In this subsection we demonstrate the main functionality of the implementation using a series of screenshots covering the life cycle of an entity. These screenshots are taken while communicating with the sensors in iMinds demo box. Figure 12 shows a screenshot demonstrating the result of sending a CoAP POST request to the EM to create an entity with five heterogeneous members. This request results in the creation of the entity with the URI “/2” and in the validation of this entity by querying all members profiles. All complexity related to the creation and validation of the entity is hidden for the client and managed transparently by the EM. At this moment, the entity has been created and the client can use the newly created entity and interact with it by sending a single CoAP request to the entity resource.

**Figure 13.**
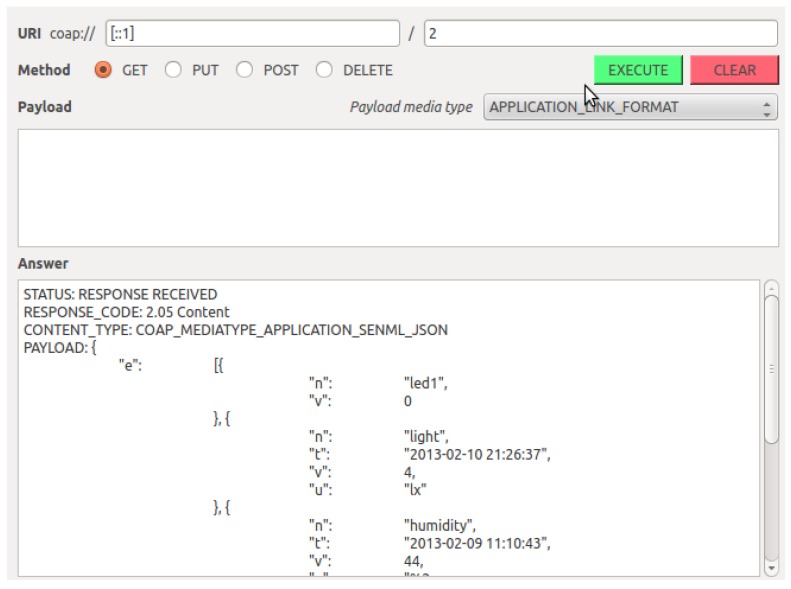
Sending a CoAP GET request to the entity results in a reply that combines the results of querying all members in the entity.

Figure 13 shows a client issuing a CoAP GET request to the newly created entity on the EM. The request ultimately results in a single reply from the EM, which combines the results of querying all five members of the entity. The client does not have to bother executing all individual requests and processing the corresponding results.

**Figure 14.**
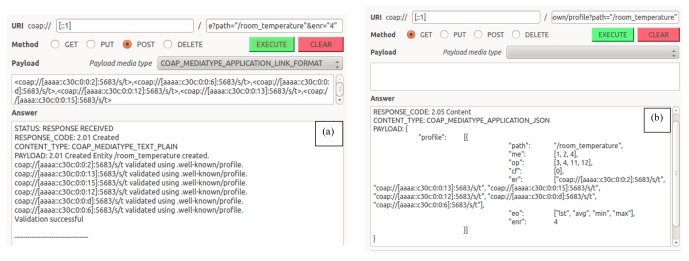

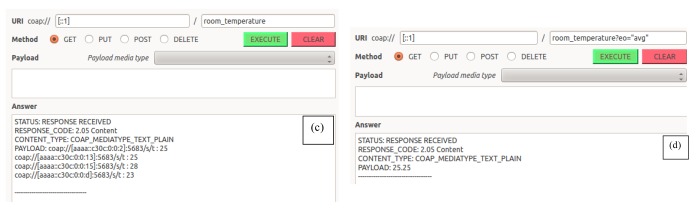
Advanced EM features. (**a**) Creation of an entity of 6 members and naming it “room_temperature” and specifying that only 4 out of the 6 members need to reply, before sending the combined reply to the client; (**b**) The profile of the newly created entity; (**c**) Querying the entity with default operation “List Replies”; (**d**) Querying the entity and specifying that the reply should only contain the average of the member values.

The above example demonstrates how an entity can be created and used with default values, since the client did not specify anything about its behavior neither at creation time, nor at usage time. However as described in Section 5, the EM allows the client to customize the behavior of the entity at creation as well as at usage time by using URI queries in the CoAP requests. Some of these features are shown in the example in Figure 14. In Figure 14a the client used URI queries at creation time to create an entity of six members, naming it “room_temperature” and specifying that only four out of the six members need to reply before sending the combined reply to the client. Figure 14b shows the profile of the newly created entity, which, among others, shows that the entity supports four entity operations (eo=[“lst”,”avg”,”min”,”max”]) with lst being the default operation as it is the first operation listed. As expected, when querying the entity, the Entity Manager returns a list of the first four replies it has received from all members in a single JSON reply in Figure 14c. When the client uses the URI query (?eo=”avg”) to obtain the average instead of the default list, the individual responses are processed by the Entity Manager and the average value is returned as shown in Figure 14d.

**Figure 15.**
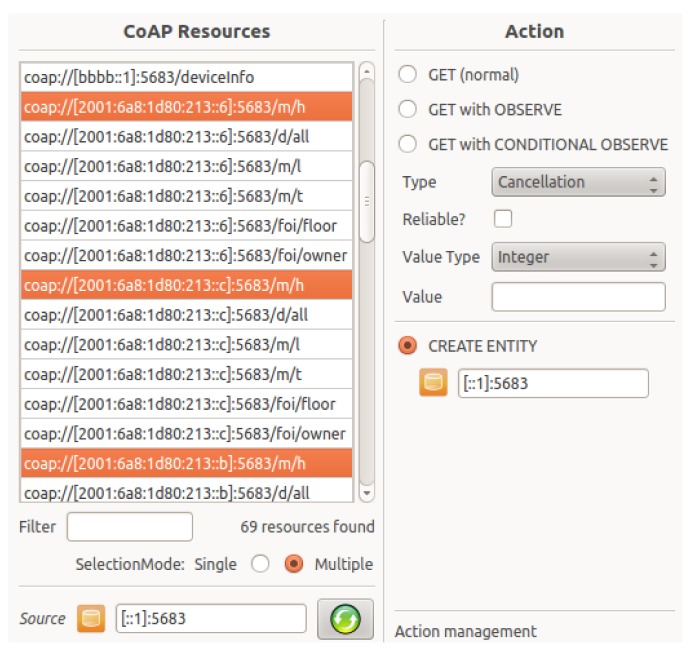
Creation of an entity by selecting three members from a list of resources provided by a resource directory.

In the last screenshot (Figure 15) we show how a client can select resources from a list of resources obtained from a resource directory to create an entity. The resource directory lists all CoAP resources of the sensors in our real-life wireless sensor network testbed, namely w-iLab.t [35]. Once the Entity Manager creates the entity, the resource directory is immediately informed about the newly created entity resource and uses this information to update the list of available resources.

### 6.3. Performance Evaluation

In order to evaluate the performance of our group communication solution and compare it with multicast based solutions we performed a series of tests using the Cooja network simulator. In this subsection we present the results of these tests and analyze key performance indicators for both approaches.

#### 6.3.1. Experiment Setup

**Figure 16.**
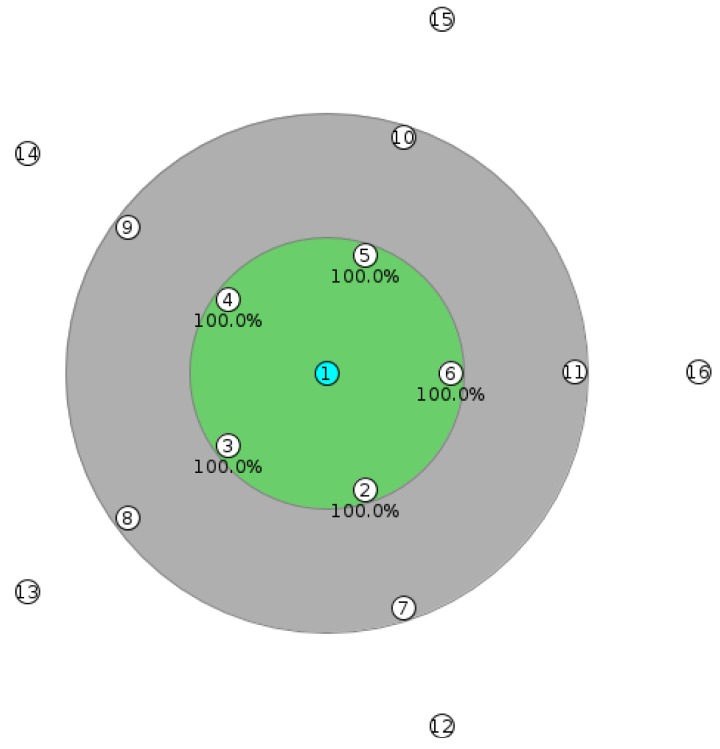
The network topology used in performance evaluation experiments. The inner circle shows the transmit range of the gateway and the outer circle shows its interference range.

In our test we used a star topology with the gateway (node ID 0) in the middle of the five-leg star (Figure 16) and the nodes along the legs at 50 m distances. The transmission range (55 m) is enough to make the signal travel from one node to the other node on the same leg, but does not allow the signal to be heard between nodes on different legs. The interference range is 105 m, so that it covers the distance between three nodes on the same leg. The reason for selecting this topology is that it allows minimizing the impact of the underlying routing protocol on our measurements, as each node has only one deterministic route to the gateway. The gateway is running the example rpl-border-router provided by Contiki and therefor it is the RPL DODAG root, delegates the global IPv6 prefix and routes traffic to and from the constrained network. All other nodes run the Erbium server extended with resource profiles and multicast support as discussed in Section 6.1. Table 1 summarizes the parameters of the simulations in Cooja.

Since evaluating the performance of MAC and routing protocols is beyond the scope of this work, we had to take special care during the experiments to make sure that what we are observing is not a result of routing or MAC errors. For example, when introducing link errors in the simulations, routes can get lost or can be changed which has a considerable impact on the delivery rate of packets in the LLN. One solution would have been to use static routes. This is however not practical, since each node needs to be programmed with its own routes each time we change anything to the topology. To avoid such manual reconfiguration and since a stable version of RPL is available within Contiki, we used RPL as a routing protocol in all of the experiments. However, in order to minimize route changes from impacting our results we have taken the following two measures in all experiments:

(1) Before sending any CoAP messages in the LLN, we wait for some time to allow RPL to establish routes to all nodes. In our chosen topologies, waiting for 2 min was in most cases sufficient to achieve this goal. In a few cases (with a high percentage of packet loss) we had to wait more than an hour, since the time between RPL neighbor updates is exponential up to a certain value.
(2) The Contiki implementation of RPL relies on Contiki to maintain the neighbor table. Contiki in turn removes a neighbor if it does not hear its heartbeat a consecutive number of times (three by default). In very lossy networks (such as in our experiments with high packet loss) this behavior might lead to neighbors being removed and thus all routes via that neighbor as well. However in all of our experiments the nodes are static and never disappear from the network. As such they should not be removed from the neighbor table. To achieve this goal we have changed the Contiki configuration parameter UIP_CONF_ND6_MAX_UNICAST_SOLICIT from its default value of 3 to 100. This allowed all experiments to be completed without routes to the nodes being lost.

With these two measures in place it was possible to get stable routes during the experiments. The other factor that may heavily impact the measurements is the used MAC protocol. In order to save energy, MAC protocols for LLNs use Radio Duty Cycles (RDC) to shut down their radios when not in use. Contiki has its own MAC protocol, called ContikiMAC, with a good RDC schema. However ContikiMAC requires more resources than other MAC protocols. In our experiments we also need, next to CoAP and multicast, debug information in order to be able to collect measurement statistics. As such, it was impossible to fit CoAP, multicast, RPL, debug info and ContikiMAC on the used Z1 motes. Instead of ContikiMAC we therefore used null-rdc, which is Carrier Sense multiple Access (CSMA) MAC protocol without RDC (radio always on). While using null-rdc is not realistic for battery-powered devices, it still helps avoiding delays in the collection of measurement statistics as imposed by the RDC protocols. However, to still get an idea about the impact of RDC on our solution, we repeated some of the tests using Xmac. Xmac uses a simple RDC, but has less stringent requirements in terms of memory consumption than ContikiMAC.

In all the experiments presented in this subsection the multicasts were sent using none-confirmable CoAP messages as required by the group communication draft [14] and the unicast were sent using confirmable CoAP messages to achieve reliability.

#### 6.3.2. Congestion Control Optimizations

An important aspect of group communication is congestion control, especially in LLN where resources are limited. Network congestion can lead to extended response times and significant energy consumption, due to frequent retransmissions of packets. CoAP provides basic congestion control by using the exponential back-off mechanism (Section 2.1) and by limiting the number of open requests from a client to any server to one request by default. Furthermore, CoAP specifies that, when using multicasts, a certain random delay should be inserted before forwarding the request to other nodes. In CoAP terms, this delay is called Leisure. The server could either use a default value for Leisure or compute a value for it. If the server has a group size estimate G, a target data transfer rate R and an estimated response size S, a rough lower bound for Leisure can then be computed as:

(2) $$\text{Leisur}e_{\text{lowerbound}}=\frac{S*G}{R}$$

When only taking into account the 1-hop neighbours of the gateway in our test network in Figure 16, G equals 5, S equals approximately 80 bytes, and the target rate can be set to a conservative 8 kbit/s = 1 kB/s. The resulting lower bound for the Leisure is then equal to 0.4 s. However, since CoAP servers will often not be able to compute the Leisure, we elected to use the default Leisure value (5s) in all of our multicast experiments. For a more complete discussion of the Leisure period and its estimation we refer to Section 8.2 of [5].

CoAP does not specify a congestion control mechanism when a single client is communicating with many servers using unicasts as is the case in our group communication solution. However our experience shows that this can quickly lead to congestion. A simple solution for avoiding network congestion when using unicasts is to limit the rate at which requests are sent. In order to examine this, we conducted a series of experiments to query an entity of five members and measure the response time, which is expressed as the time between the moments the client issues the request to the EM until it gets back the response. We repeated the same experiment for different delays between the requests sent from the EM to the members. We repeated the experiment 50 times for each setting and computed the averages. The same set of experiments was repeated when all members were either one or two hops away and while using null-rdc or Xmac.

**Figure 17.**
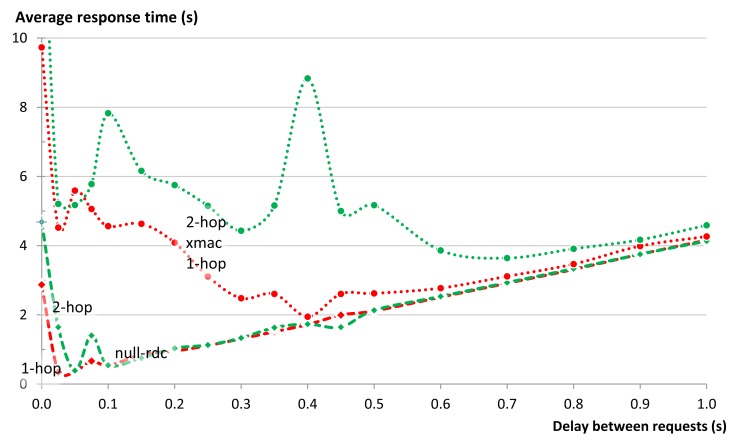
The average response time of an entity can be improved by inserting small delays between the requests for individual entity members. The best delay depends on the topology and the used MAC protocol.

As expected, the experiments revealed that the average response time of an entity can be improved by inserting small delays between the requests for individual entity members (Figure 17). However, the best delay depends on both the topology and the used MAC protocol. This is most obvious in the graph of Xmac for 1-hop communication. Here one observes two peaks at about 100 ms and 400 ms. The first peak is a result of collisions between the forwarded requests from the nodes at the first hop to the nodes at the second hop with the delayed requests for the next member. The second peak is a result of collisions between the replies from the members at the second hop with the delayed requests for the next member. For delays less than 0.5 s the used MAC protocol had more effect on the response time than the delay inserted between the requests. However as the delay between requests grows larger it becomes the dominating factor for the total response time with a linear relationship between the two.

In the remaining experiments we used only null-rdc as MAC protocol for two reasons. First, we wanted to avoid measuring the delays in communication as imposed by the use of Xmac. Second, Xmac does not support multicast well, and thus we would not have been able to make a fair comparison between our group communication solution and multicast based solutions.

For the star topology which we used in most of our experiments, a delay of about 50 ms provided the best response when null-rdc was used. As a result we have used a 50 ms delay between requests to the members in all other experiments discussed later in this section. In addition to this delay there are other delays that impact the communication. The nodes and the EM need some time to process the CoAP packets they send and receive (e.g., time needed by nodes to prepare the CoAP reply and the delay needed by the EM to combine all replies into one reply to the client). We call the sum of all such delays the Processing Delay *D_p_*. Finally we call the sum of the average signal propagation time and the average time need to send and receive a packet over any transmission link the Transmission delay *D_t_*, We have experimentally evaluated *D_p_* and *D_t_* by averaging the values for 100 transmissions for our network topology when communicating with the nodes that are only 1-hop, 2-hops or 3-hops away. Table 2 provides a summary of the delays in our experimental topology.

#### 6.3.3. Reliability

Reliability is a key performance indicator. In this subsection we first present the theoretical model for calculating the reliability of the two group communication approaches and then present the results obtained from our simulations.

##### Theoretical Calculation

Let us assume that the probability of losing a packet when it is sent over any link in a lossy network is equal to *l*. Thus the probability for success at any link-transmission in the network equals 1 − *l*. If the communication is over N-hops, the number of links equals *N* and the probability that the communication succeeds over the 2*N* link-transmissions (since every link has to be crossed once for the request and once for the reply) is:

(3) $$P_{N_{\text{non}}}={\left(1-l\right)}^{2N}$$

This communication success probability applies for multicast communication as well as for non-confirmable (non) CoAP unicast communication. However, when using confirmable (con) CoAP unicast communication as the case in our group communication solution, CoAP tries to achieve reliability by using a simple stop-and-wait retransmission with exponential back-off (see Section 2.1). This means that if a reply to a confirmable packet is not received within the back-off time, the CoAP sender will retransmit the packet. If a reply to the first transmission is not received, CoAP will retry the transmission until MAX_RETRANSMIT (by default 4) is reached. Again these retransmissions have the same probability for success as the first attempt. And thus the probability that *r* retransmissions are needed for successful transmission over *N* hops (*r* can go from 0 to 4) is:

(4) $$R_{N_{r}}=P_{N_{\text{non}}}{\left(1-P_{N_{\text{non}}}\right)}^{r}$$

Thus the probability for success when using CoAP con communication equals the sum of the probabilities of successful communication of any of the five transmission attempts:

(5) $$P_{N_{\text{con}}}=\sum_{r=0}^{4}R_{N_{i}}=1-{\left(1-P_{N_{\text{non}}}\right)}^{5}$$

In many group communication use cases, it is desirable to get answers from all members of the group. A complete group communication is considered successful when all members in the group also have successful communication:

(6) $$\begin{array}{c} P_{NG_{non}}={\left(P_{N_{non}}\right)}^{G}\\ P_{NG_{con}}={\left(P_{N_{con}}\right)}^{G} \end{array}$$

This reliability of the unicast group communication is achieved by relying only on default CoAP retransmissions. If higher reliability is desired, the EM can perform its own retransmissions or fine-tune the default CoAP retransmission settings on an entity-wide level or per member.

##### Experimental Evaluation

**Figure 18.**
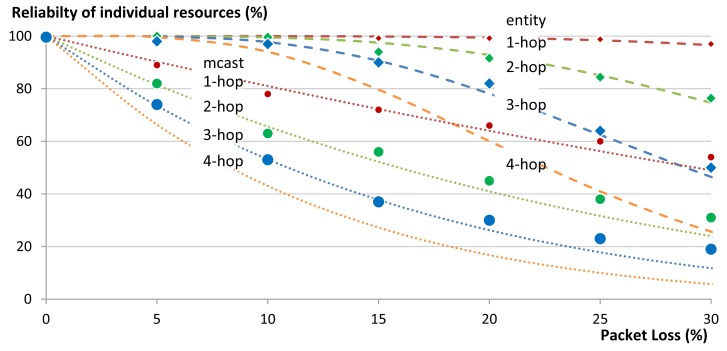
The reliability of individual group members is a lot better when using entity based group communication.

To measure the reliability we used the same star topology to communicate with a group of five members that were either 1-, 2-, or 3-hops away from the gateway. We varied the percentage of packet loss in the network in 5% steps and measured the reliability of getting responses to the respective requests. We repeated the same experiment for our group communication solution and for multicasts. We run each experiment 50 times. Figure 18 shows the effect of packet loss on the communication reliability in our 1-, 2-, and 3-hop star network. Multicasts are not transported reliably and thus reliability of the network decreases as soon as there is packet loss in the network. When using our unicast group communication solution, CoAP confirmable messages are used. In our 1-hop test topology reliability was higher than 99% even when the packet loss of the network reached 25%. At 30% packet loss the reliability is reduced to 97% (compared to 49% in the case of multicasts). Figure 18 also shows that the packet loss increases with an increasing hop count, both for unicast and multicast communication. This is due to the fact that every message (both request and reply) between a client and a server has an additional chance of getting dropped at each hop on the way to its destination. Nevertheless, in our 2-hop network 100% reliability was maintained for unicast communication until a packet loss ratio of 10%. In the 3-hop network the unicast reliability started dropping below 100% already by 5% packet loss. The dashed and the dotted lines in Figure 18 are the theoretically expected values up to networks with 4-hops, while the points are the actual obtained results from the experiments (up to networks with 3-hops). It is clear that there is a good match between both.

**Figure 19.**
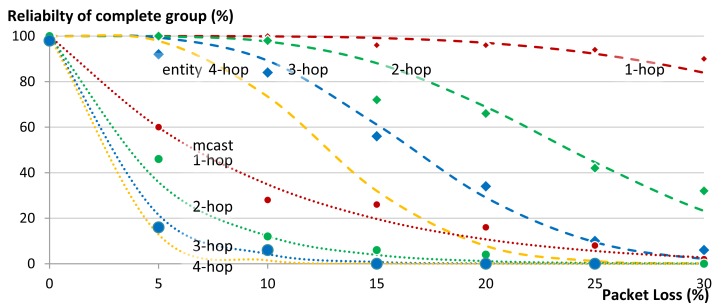
The reliability of the complete group is less than the reliability of individual members (Figure 18). Again, the reliability of the complete group is a lot better when using entity based group communication.

Figure 19 shows the effect of packet loss on the reliability of the complete group in our 1-, 2-, and 3-hop star network. Certainly the reliability of a complete group is less than the reliability of its individual members, since the loss of a message to or from a single member, renders the complete group request unsuccessful. In these cases the use of multicasts does not provide good results. Already at 5% packet loss the reliability of a 1-hop network drops to 80% and below 20% for a 3-hop network. In contrast, our unicast based group communication maintains 100% reliability in the 1-hop network and only drops to 92% in the case of 3-hop network. Figure 19 shows good match between the theoretically expected values (the lines) and the actual obtained results from the experiments (the points).

#### 6.3.4. Number of Packets

Another key performance indicator is the amount of energy consumed by the network to complete the communication task. The main two contributing factors to the energy consumption are the efficiency of the RDC and the numbers of packets sent. The RDC mostly depends on the MAC protocol and is beyond the scope of this article. We use the number of packets sent inside the LLN as an indicator for the amount of energy consumed by the network as exact numbers for energy consumption are hardware dependent. In this subsection we first present the theoretical model for calculating the number of packets and then present the results obtained from our simulations.

##### Theoretical Calculation

When using multicast group communication, the number of packets that are sent depends on the topology of the network. In our star topology, all nodes that are one hop away can be reached with just one multicast packet. After the first hop, the paths are not shared by any node and the packets have to travel similar to unicasts along them. When the requests reach their destinations, replies are generated and are sent back using non-confirmable unicast messages. Thus, in the case of a successful communication, the number of transmitted packets can be obtained as follows:

(7) $$\text{pkt}s_{N_{\text{sucess}}}=1+G(2N-1)$$

Taking into account the probabilities of failure at each link-transmission (see Section 6.2.3), the average number of transmitted packets when using multicast group communication can be obtained as:

(8) $$\text{pkt}s_{N_{\text{mcast}}}=(\sum_{i=0}^{2N-1}l{\left(1-l\right)}^{i}\times (iG+1))+{\left(1-l\right)}^{2N-1}\times (1+G(2N-1))$$

This formula takes into account the fact that the request or reply can get lost at any intermediate hop (total of *i* = 0, …,2*N* − 1 possibilities with the probability *l*(1 − *l*)*^i^*), resulting in a different number of packets being transmitted (*iG*+1).

When using our group communication solution, all transmissions are unicast and thus in the case of a successful communication, the number of transmitted packets can be obtained as follows:

(9) $$\text{pkt}s_{N_{\text{sucess}}}=2NG$$

And the average number of packets in case of failure (*i.e*., packet loss somewhere on the path) as:

(10) $$\text{pkt}s_{N_{\text{failure}}}=\{\begin{array}{cc} \frac{G\sum_{i=0}^{2N-1}l{\left(1-l\right)}^{i}\times (i+1)}{\sum_{i=0}^{2N-1}l{\left(1-l\right)}^{i}}, & l>0\\ 0, & l\leq 0 \end{array}$$

The formula is normalized as the sum of all probabilities in the nominator is not 1 since it does not include the probability of success over all links *P_N_*. To avoid division by zero we set *pkts_N_failure__* = 0 when *l* ≤ 0.

And the average number of transmitted packets in these cases of retransmissions can be calculated as:

(11) $$\text{pkt}s_{N_{r}}=r\times \text{pkt}s_{N_{\text{failure}}}+\text{pkt}s_{N_{\text{sucess}}}$$

If the last retransmission attempt fails, the sender is notified about the failed transmission. Thus the probability for failure for an N-hop communication:

(12) $$R_{N_{\text{failure}}}={\left(1-P_{N}\right)}^{5}$$

And the average number of transmitted packets in this case can be calculated as:

(13) $$\text{pkt}s_{N_{5}}=5\times \text{pkt}s_{N_{\text{failure}}}$$

Thus the average number of transmitted packets for N-hop confirmable CoAP unicast communication can be calculated as follows:

(14) $$\text{pkt}s_{N_{\text{entity}}}=\left[\sum_{r=0}^{4}R_{N_{i}}\times \text{pkt}s_{N_{r}}\right]+R_{N_{\text{failure}}}\times \text{pkt}s_{N_{5}}$$

##### Experimental Evaluation

**Figure 20.**
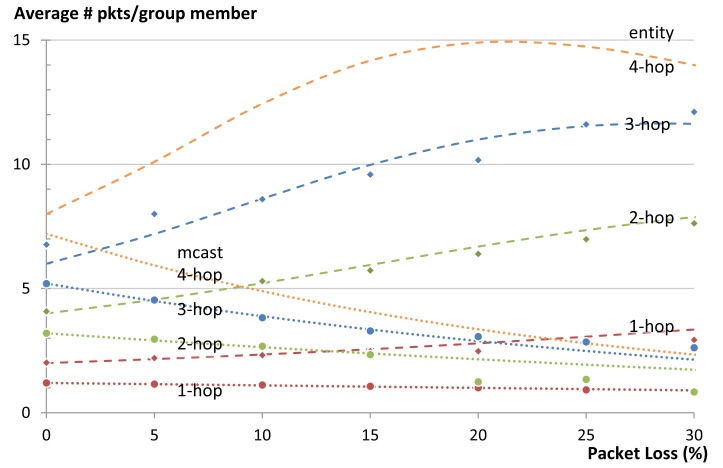
The number of packets sent in the LLN for each group member at different packet loss levels. For multicasts the number of packets decreases as the loss increases. For unicasts the number increases due to CoAP retransmissions.

When multicasts are used, one request is sent to multiple destinations, and one reply is sent by each member. For our five nodes that are 1-hop away, assuming there is no packet loss in the network, the number of transmitted packets is thus six packets (an average of 1.2 packets/member). When the network is lossy the number of packets can only become less, as packets are being dropped. This can be clearly seen in Figure 20 for 1-, 2- and 3-hop networks. On the other hand, when using unicasts the number of packets increases as the loss increases. This is a logical result of packets being retransmitted by CoAP to compensate for the packet loss. The dashed and dotted lines in Figure 20 are the theoretically expected values, while the points are the actual obtained results from the experiments. It is clear that there is a good match between both.

#### 6.3.5. Response Time

**Figure 21.**
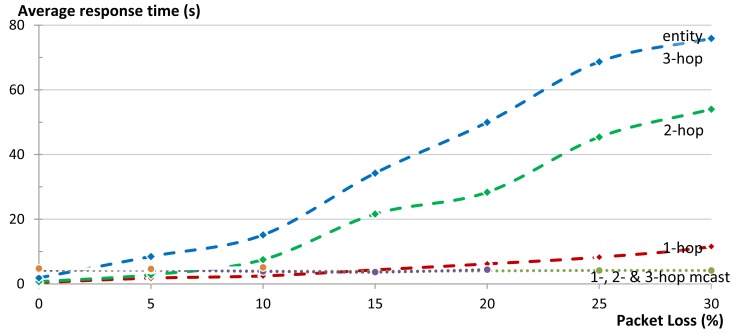
Average group response time.

Another key indicator of the performance of any group communication solution is its response time. Figure 21 shows that the average response time when using our group communication solution increases with the increase of packet loss in the network. When there is no loss, both group communication methods have similar response times (0.7 s for multicasts and 0.8 s for our solution). When the packet loss increases, the response times for multicasts remain constant, since either the packet is delivered on time or it is just dropped. In the case of our solution, when a packet is dropped CoAP attempts to retransmit it, leading to an increased overall response time. At 15% packet loss, the average response time for the 1-hop network is about 3.5 s and increases to about 10 s at 30% packet loss.

**Figure 22.**
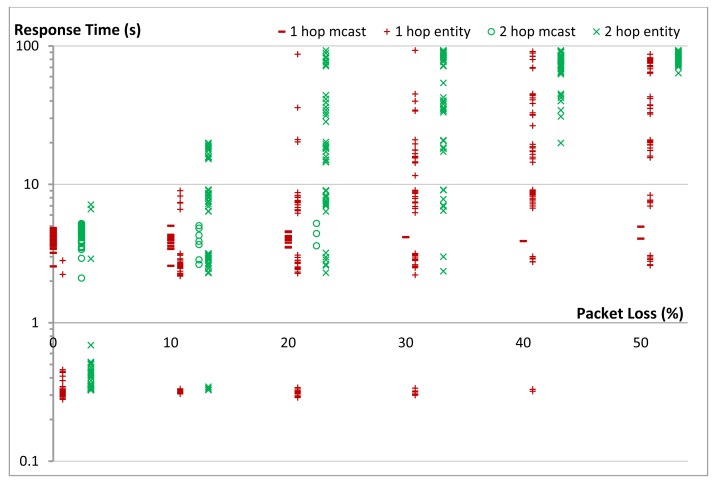
Scattergram of the response times for 1- and 2-hop networks using multicast and unicast group communication for different packet loss values.

The CoAP retransmission behavior can be clearly seen in Figure 22, which shows the same results of Figure 21 in the form of a scattergram. It shows how the response times of the unicasts are grouped along the time axis. Replies with a response time of around 1 s were sent without any retransmissions. Replies with a response time around 7, 15 and 30 s occur when one, two or three retransmissions take place respectively. The last group between 60 and 100 s is for replies that were received after four retransmissions, or those that timed out without a reply.

An interesting aspect to consider is the impact of long delays in network on the client. In our case both the communication between the client and EM from one side and the communication between the EM and the members on the other side are using the same CoAP time out mechanism with default values. Thus it is expected that the client might time out and start retransmitting the request before the EM requests to the members are answered or have timed out. To avoid these unnecessary retransmissions from the client, the EM responds to the client with an empty acknowledgment message as soon as it receives the request, indicating that the EM is processing the request and will send a separate reply once an answer is ready. As a result the client does not send any further retransmissions of the request.

#### 6.3.6. Impact of Caching

As mentioned in Section 2.1 CoAP foresees a freshness model for responses. A CoAP server may include a Max-Age option in the reply to indicate the maximum time a response may be cached before it is considered not fresh. If this option is not included in any CoAP response, it can be assumed that the response will be fresh for 60 s and thus will not be queried again by a cache within this time frame. Max-Age values in responses should take into consideration the frequency at which the value of the corresponding resource changes. By doing so, unnecessary queries to constrained devices can be heavily reduced, especially for resources that do not change frequently.

When a client makes a multicast request, the cache always makes a new request to the multicast group (since there may be new group members that joined meanwhile or ones that did not get the previous request) (see Section 8.2.1 of the CoAP draft [5]). So, the main problem is that the cache is not aware of all receivers in a multicast group. This information is needed in order to be able to verify whether a response for all receivers in the multicast group has been cached. If the cache knows this information (which is not the case for a normal cache), the cache could serve the multicast request. But even then the multicast has to propagate in the network as soon as one of the responses is missing.

**Figure 23.**
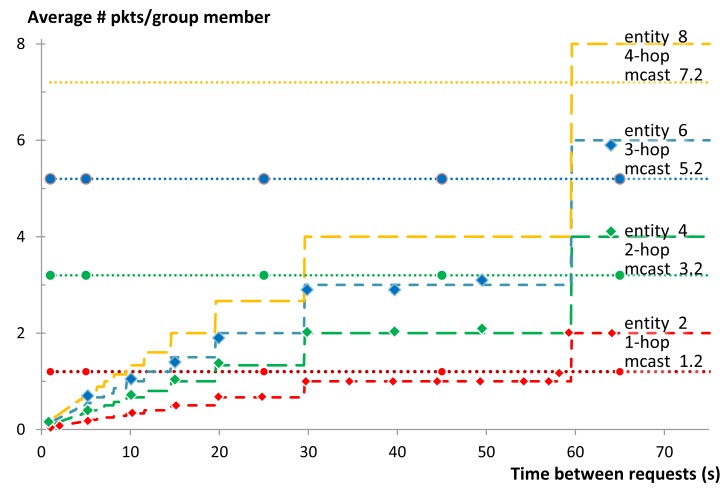
When requests are served from the cache the number of packets transmitted inside the LLN is reduced.

In order to test the behavior of both group communication approaches when caches are used, we have conducted a series of tests using our star topology in its 1-hop configuration with five members. In the tests we varied the time between requests to the group in 5 s increments and measured the number of packets transmitted inside the LLN and the response time. We sent 60 requests per experiment. The experiments were conducted without introduction of any packet loss. The average number of packets transmitted inside the LLN is shown in Figure 23. As expected when using multicasts, regardless of the frequency at which requests are sent, there was always one request and five replies from the five members resulting in an average value of 1.2, 5.2 and 7.2 packets/member for group members that are 1-, 2- and 3-hops away from the EM respectively. On the other hand, when using our unicast based solution the average number starts at 0.04, 0.07 and 0.1 packets/member and increases in steps to reach two, four and six packets/member for group members that are 1-, 2- and 3-hops away from the EM respectively. The reason for this behavior is that the initial requests are always sent to all members as the cache is still empty. Since the replies in our example did not include the Max-Age Option, the cache assumed that the replies were valid for the default value of 60s. Subsequent requests were thus served from the cache as long as the first replies were still considered fresh. Once the Max-Age value expired new requests were sent to the members again.

**Figure 24.**
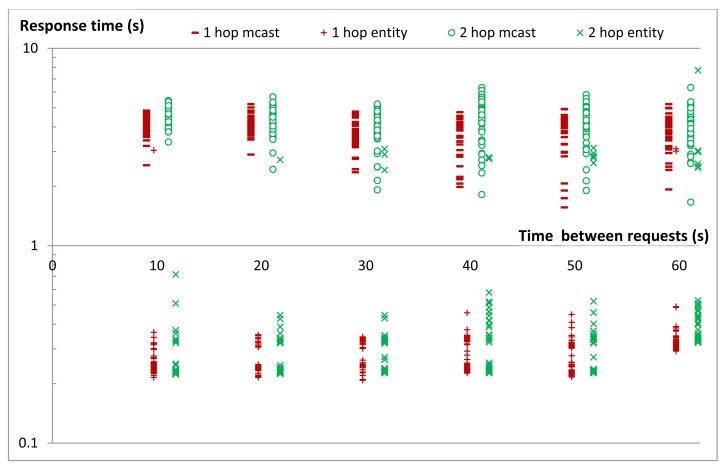
Responses that are served from the cache are much faster than responses obtained from the nodes inside the LLN.

Figure 24 shows the response times for the requests for both group communication approaches, when a cache is used for the cases in which the group members were 1- and 2-hops away from the EM. For multicast the response time is between 2 and 6 s regardless of the delay between requests. In the case of our group communication approach one can see that there are two groups of replies. The first group is between 0.20 and 0.25 s and the second group is higher than 0.3 s. The first group represents the replies that were served from the cache, while the second group represents the replies that were not fresh in the cache and thus obtained from the group members. With the increase of the time between the requests, one can observe how the number of replies served from the cache decreases. When the time between requests becomes larger than Max-Age, the number of requests served from cache becomes zero.

The cache stores the individual replies from the group members. This makes it possible to benefit from the cache even when different clients request a different Entity Operation. For example if two clients access the same entity within Max-Age, but one is requesting the average and the other is requesting the maximum value, then each entity member will be queried only once. The EM then processes the replies from the members and the cache and assembles the reply to the client as was requested. The same applies if a single member is part of two different entities (overlapping entities). The member will be queried only once within Max-Age, regardless of the entity that is requesting it. Finally, the cache can be populated via other requests/responses that pass via the gateway; it's not limited to just the requests/responses employed by the EM.

Please note that caching is very interesting, but it will not work for actuators. However, for actuators one probably wants high reliability, which is again in favor of our solution.

#### 6.3.7. Entity Validation Overhead

As described in Section 5.3, the EM can perform a validation process on the entity at creation time. In this subsection we measure the communication overhead of this validation process as a function of the number of group members. For this, we need a topology in which all group members are located at the same distance (in hops) form the Entity Manager. Otherwise the results will be influenced not only by the group size, but also by the hop count. Therefore, we use a single hop topology consisting of several nodes in a single collision domain. Every node in this LLN had exactly one resource that is part of the entity. This topology minimizes the effect of routing and forwarding delays on the validation process. For this topology, we performed several experiments and measured the number of packets sent inside the LLN during the validation process and the time that was needed to complete the process. We did not utilize the cache in this experiment. The experiment was conducted for several entity sizes; starting with a single member entity up to an entity with 24 members. The experiment was repeated for several packet loss percentages between 0% and 20%.

**Figure 25.**
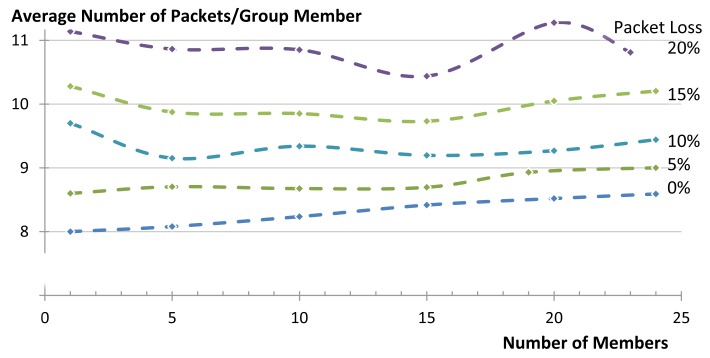
The number of required packets needed to validate a single entity member increases slightly (due to CoAP retransmissions) with the increase of packet loss in the LLN. As a minimum eight packets are required to obtain the profile of the respective member.

In order to validate an entity member, the EM starts by querying the profile of that resource. In our case the profile was 107 bytes long. A minimum of eight packets was required to obtain it (Figure 25). The reason for this is that the maximum usable payload size in our LLN is about 60 bytes (see Section 2.3). This means that blockwise transfer should be used to obtain the profile. As mentioned in Section 2.3, CoAP utilizes predefined block sizes. Unfortunately we cannot use the block size of 64 (since it is just above the usable payload size) and have to use 32-Byte blocks. Consequently, four packets are needed to obtain the complete resource profile and another four packets to acknowledge the receipt of the requests and transfer the profile parts. Figure 25 shows that in the simplest case (one member in the entity and no packet loss) exactly eight packets were needed. When the number of members in the entity increases, so does the average number of required packets. This increase is due to the fact that the probability of packet drops due to collisions increases with the increase of the collision domain size. Since we are using confirmable CoAP messages, CoAP utilizes its retransmission mechanism to obtain the missing packets. Similarly, if we inject random packet loss in the network more packets will be transmitted to compensate for the loss. In addition to the CoAP retransmissions the EM uses its fallback mechanism for validation. This means that for cases with a lot of packet loss, it may happen that all retransmission attempts to obtain the profile fail. In these cases the EM tries to check if the resource is available based on the content of /.well-known/core of the node. Here again, blockwise transfer is used to obtain the information in 16 packets (eight requests, eight replies). If this also fails, the EM tries to query the resource to check if it exists, consuming two packets (one request, one reply).

**Figure 26.**
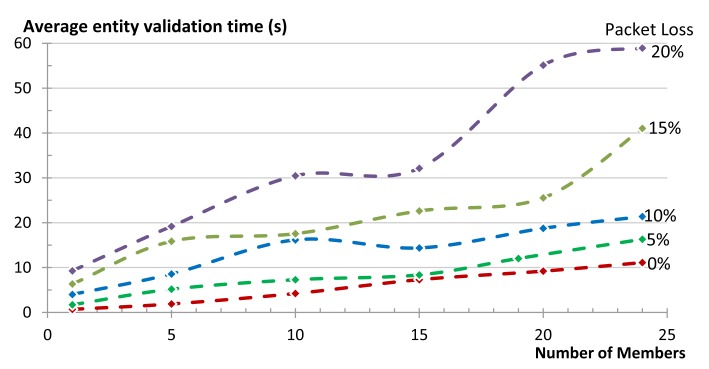
Average entity validation time.

As expected, Figure 26 shows that the average entity validation time increases when the number of members in the entity is increased since validation requests are sent sequentially. The validation time also increases with the increase of packet loss in the LLN. The increase here has more profound effect on the delay since CoAP doubles the timeout between retransmissions, explaining the exponential trend in the delay.

Certainly, the entity validation overhead has negative impact on the energy consumption of the nodes in the LLN. If an entity will be created and used for a very limited time, this impact cannot be ignored. However, in many use cases it is expected that, once created and validated, entities will have a long life span. For example an entity that represents the lights in a certain room will change only if the layout of the room and lights change, which is in many cases a relatively rare occasion. In these cases the communication overhead of validation can be ignored. Additionally, one should also consider that the information needed for validation (profile; .well-known/core) is fairly static and thus can be efficiently cached by having a very-long max-age value. In such cases the validation can be based on the information obtained from the cache and thus be done almost immediately, without the need for packet transmissions inside the LLN.

## 7. Discussion

In this section we look again at the requirements for the IoT scenarios as presented in Section 3 and discuss how our solution addresses these requirements. We also provide the drawbacks of our solution in light of these requirements and the insights gained from experimental evaluation of our solution.

(1). *Flexibility*: our CoAP group communication solution is highly flexible when compared to other solutions. Other than supporting the standard base CoAP protocol, group members do not have to support any additional extensions. Since group definition and behavior are set on the Entity Manager only, changes and extensions can be done on the Entity Manger without the need for any changes on the individual members. We support homogeneous and heterogeneous entity in terms of their CoAP resources. A group can be built from members as along as the have a shared subset of CoAP methods. Members do not have to offer the exact resource name or the same content-type, since the Entity Manger can take care of conversion when possible. Since we use IPv6 unicasts for communication between the Entity Manager and the members, the members can be located anywhere on the IPv6 Internet as long as they are reachable.
(2). *Light-Weight (footprint)*: since our solution does not require any additions to standard CoAP implementation, it can run on Class 1 devices as already demonstrated in the three CoAP plugtest events. Furthermore the members do not store any information about which groups they are part of. Thus our solution scales well with the number of groups a certain member can be part of.
(3). *Use of Standards*: our Solution is based on CoAP, which is expected to become the application layer standard the IoT. We rely only on the base CoAP standard and require no optional extensions to it. However, if available, the resource profile extension can be used to better understand the capabilities on the individual members in order to apply advanced features such as content type conversions.
(4). *Performance*: as our solution uses only standard CoAP over unicast UDP/IPv6 it is a cache friendly solution. Caches are a powerful tool in reducing the number of messages inside LLNs and enhancing response times. However only CoAP GET method can be cached and thus no benefit of caches can be expected for the other CoAP methods (*i.e.*, GET for actuator data). Nevertheless, in these cases it is often more important to reliably deliver the message even if performance is reduced as a result.
(5). *Validation and Error Handling*: we support heterogeneous members that might have properties that cannot be combined. Thus whenever a group is created the Entity Manager tries to validate the group in order to make sure that the group works as intended and informs the creator about the validation results. By giving the possibility to build the reply from just a subset of the members, the group becomes tolerant in handling certain error conditions such as node or route failures.
(6). *Reliability*: by using CoAP confirmable messages we rely on CoAP retransmission mechanisms to handle reliability of communication with the entity members. In addition it is possible for the EM to customize the parameters for retransmissions (number of retransmissions and time-outs) for all or certain group members based on the history of communication with these members.
(7). *Ease of Group Manipulation*: if the needs of the user change with time, our solution enables changing group memberships based on these needs by just changing the entity definition on the EM. The group members are not involved in this change and do not need to be reprogrammed or reconfigured in any way. By integrating the resources of the members and the Entity Manager into a resource directory, it becomes easy to locate and combine resource into new entities. This can benefit both machine-to-machine resource discovery as well as end user using Graphical User Interfaces to browse and build new entities.
(8). *Expressiveness/Control*: by supporting processing of individual result at the EM, we enable greater control over the results that are sent to the user as a result of contacting the group members. Currently we support sending all replies as a list, or combining the results in one arithmetic value (minimum, maximum, or average). We also support the user to specify the number of members that should reply before an answer is composed and sent back to the requester. Additional features to improve the control over entity behavior can be easily added by extending the EM functionality without the need to extend the members themselves.
(9). *Security*: by using only standard CoAP unicast messages for communication, we make it possible to apply whatever used transport security mechanisms (such as DTLS) to these messages as well.

It is clear from the discussion above, that our solution is capable of addressing the requirements we have put forward for group communication in IoT scenarios. Of course, the gain in flexibility comes with some drawbacks as well. Compared to a multicast solution, a unicast-based approach in general leads to more packets and to increased latency, with the exact values strongly depending on the topology. Of course, the experimental evaluation also reveals that a multicast-based solution is inferior in cases where packet loss cannot be tolerated, caching is relevant, security is required or heterogeneous groups need to be supported.

## 8. Conclusions/Outlook

In this paper we have presented a novel solution for interacting with a group of CoAP resources across multiple smart objects. It provides an interesting alternative to multicast-based solutions, which are challenging to realize in a constrained environment. It is also an alternative to application-based solutions, which simply program the required functionality. An Entity Manager, which can reside anywhere, turns groups of resources into entities. A strong point of our approach is that it nicely integrates the important aspects of entity management: creation, validation, usage and manipulation. At the side of the constrained devices, it requires no additional complexity, except optional support for profiles in order to realize more powerful validation. The introduction of entity profiles introduces a lot of flexibility and opportunities for further extensions regarding how entities should behave. We have implemented our proposal and demonstrated and validated its feasibility. We have also performed a detailed performance evaluation of our solution. We explored several aspects (overhead, timing, scalability, *etc*.) related to the creation, validation and usage in realistic sensor networks and compared it with existing multicast-based solutions. As such, we think that our solution is a powerful enabler for group communication in LLNs and an interesting building block for IoT applications.

As future work we would like to test our solution on larger test-beds and with different use-cases. We will add more intelligence to the Entity Manager to make it optimize the response time and network overhead. One such optimization could be the adaptation of the CoAP retransmission parameters for certain members based on the history of communication with these members. Also, additional ways to interact with entities, by extending the profiles, will be investigated.

## Figures and Tables

**Table 1.** Cooja network simulator settings.

**Radio Medium**	**Unit Disk Graph Medium (UDGM): Distance Loss**
Transmit Range	55 m
Interference Range	105 m
Distances between nodes	50 m
Transmit Ratio	100%
Receive Ratio	50%–100% (depending on the experiment)

**Table 2.** Summary of communication delays in used topology.

**Entity Delay between Requests to Group Members**	***D****_e_* ** = 50* ms***
Processing delay	*D_p_*=120 *ms*
Transmission delay	*D_t_*=23 *ms*

## Notes

**Authors Contributions** This paper is part of a Ph.D. Thesis written by Isam Ishaq under supervision of Jeroen Hoebeke, Ingrid Moerman and Piet Demeester. Floris Van den Abeele implemented the resource directory, Jen Rossey helped in the sensor implementation. Both have reviewed the paper and provided valuable comments for improvements.
